# The Chemical Constituents from Fruits of *Catalpa bignonioides* Walt. and Their α-Glucosidase Inhibitory Activity and Insulin Secretion Effect

**DOI:** 10.3390/molecules26020362

**Published:** 2021-01-12

**Authors:** Youngse Oh, Dahae Lee, SeonJu Park, Seung Hyun Kim, Ki Sung Kang

**Affiliations:** 1 College of Pharmacy, Yonsei Institute of Pharmaceutical Sciences, Yonsei University, Incheon 21983, Korea; oys9300@naver.com (Y.O.); sjp19@kbsi.re.kr (S.P.); 2 College of Korean Medicine, Gachon University, Seongnam 13120, Korea; pjsldh@naver.com; 3 Chuncheon Center, Korea Basic Science Institute (KBSI), Chuncheon 24341, Korea

**Keywords:** ^1^H-NMR, ^13^C-NMR, *Catalpa bignonioides*, triglucoside flavone, iridoids, α-glucosidase inhibitory activities, glucose-stimulated insulin secretion

## Abstract

Catalpa pod has been used in traditional medicine for the treatment of diabetes mellitus in South America. Studies on the constituents of Catalpa species have shown that it is rich in iridoids. In the present study, three previously undescribed compounds (**2**–**4**), including two secoiridoid derivatives along with twelve known compounds, were isolated from the fruits of *Catalpa bignonioides* Walt. In addition, fully assigned ^13^C-NMR of 5,6-dihydroxy-7,4’-dimethoxyflavone-6-*O*-sophoroside (**1**) is reported for the first time in the present study. The structures of compounds were determined on the basis of extensive spectroscopic methods, including UV, IR, 1D, and 2D NMR, mass spectroscopy, and CD spectroscopic data. All the isolated compounds were evaluated for α-glucosidase inhibitory activity. Among the tested compounds, compounds **2**, **3**, and **9** exhibited significant inhibitory activity against α-glucosidase enzyme assay. Meanwhile, the effect of compounds **2**, **3**, and **9** on glucose-stimulated insulin secretion (GSIS) was measured using pancreatic β-cells. Compounds **2**, **3**, and **9** exhibited non-cytotoxicity-stimulated insulin secretion in INS-1 cells. The expression levels of proteins associated with β-cell function and insulin secretion such as phosphorylation of total insulin receptor substrate-2 (IRS-2), phosphatidylinositol 3-kinase (PI3K), Akt, activated pancreatic duodenal homeobox-1 (PDX-1), and peroxisome proliferator-activated receptor-γ (PPAR-γ) were increased in INS-1 cells after treatment with compounds **2**, **3**, and **9**. The findings of the present study could provide a scientific warrant for their application as a potential antidiabetic agent.

## 1. Introduction

Diabetes mellitus (DM) is a metabolic disease characterized by glucose intolerance and changes in lipid and protein metabolism [1]. DM can be divided into insulin-dependent diabetes mellitus (type 1) and non-insulin-dependent diabetes mellitus (type 2). Type 2 diabetes is a metabolic disorder resulting from the body’s inability to produce, and properly utilize insulin, which causes hyperglycemia [2]. Many oral hypoglycemic agents for clinical use are available for the treatment of DM, but these synthetic agents produce severe side effects such as hypoglycemia, weight gain, gastrointestinal disturbances, and liver toxicity [3]. With the recommendations of the World Health Organization (WHO) expert committee on DM, many researchers have attempted to find more effective natural products with fewer side effects [4].

*Catalpa bignonioides* Walt. (Bignoniaceae), commonly known as a bean tree, is a traditional folk medicine in South America for the treatment of gastric diseases, helmintic infections, oncological diseases, bronchial diseases, carbuncles, scabs, and abscesses [5]. Previous phytochemical studies revealed that the extracts of *C. bignonioides* contained numerous classes of compounds such as fats, sugars, tannins, flavonoids, quinones, alkaloids, triterpenes, steroids, iridoids, and phenolic compounds [5,6]. Especially, catalpol, widely found in plants of genus *Catalpa*, has been clinically used for the management of diabetes [7]. A recent study reported the anti-inflammatory and anti-nociceptive activity of *C. bignonioides* aqueous extracts. Compared to research on the antidiabetic activity of its extract, very few studies have been conducted on chemical constituent studies and their activities of the fruits of *C. bignonioides*. A previous study verified that catalpic acid from the *Catalpa* seed decreases abdominal fat deposition, triglyceride concentrations, and glucose and insulin homeostasis, while it increases high-density lipoprotein cholesterol, and modulates white adipose tissue gene expression [8]. As a part of an investigation to find a new natural anti-diabetes source, a phytochemical study on methanol extract of *C. bignonioides* fruits was carried out. As a result, one new flavone glycoside (**2**) and two new iridoids (**3** and **4**) together with twelve known compounds were obtained. Among the known compounds, ^13^C-NMR of compound **1** was fully assigned and described for the first time in the present investigation. These isolated compounds were evaluated for α-glucosidase inhibitory activity and glucose-stimulated insulin secretion (GSIS) effect.

## 2. Results and Discussion

### 2.1. Structure Elucidation

**Figure 1:**
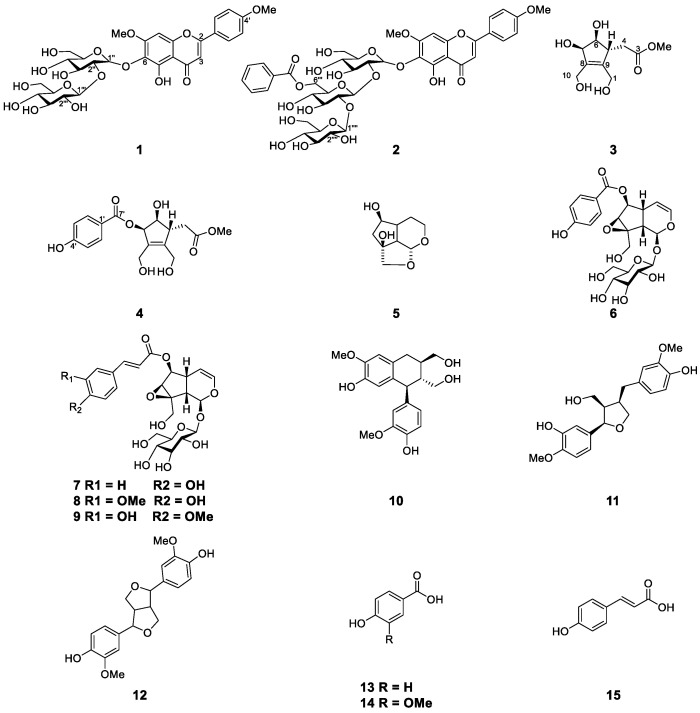
Chemical structures of compounds **1**–**15**.

Using various chromatographic resin and isolation techniques, one new flavone glycoside (**2**) and two new iridoids (**3** and **4**) along with twelve known compounds were isolated from the MeOH extract of the fruits of *C*. *bignonioides*. The known compounds were identified as 5,6-dihydroxy-7,4′-dimethoxyflavone-6-*O*-sophoroside (**1**) [9], des-*p*-hydroxybenzoyl-3-deoxycatalpin (**5**) [10], catalposide, specioside, 6-*O*-*trans*-feruloyl catalpol (**6**–**8**) [11], minecoside (**9**) [12], isolariciresinol (**10**) [13], (+)-lariciresinol and pinoresinol (**11** and **12**) [14], 4-hydroxybenzoic acid (**13**) [15], vanillic acid (**14**) [16], and *trans*-*p*-coumaric acid (**15**) [17], by comparing their physical and spectral data with that of those reported in the literature (Figure 1).

**Figure 2:**
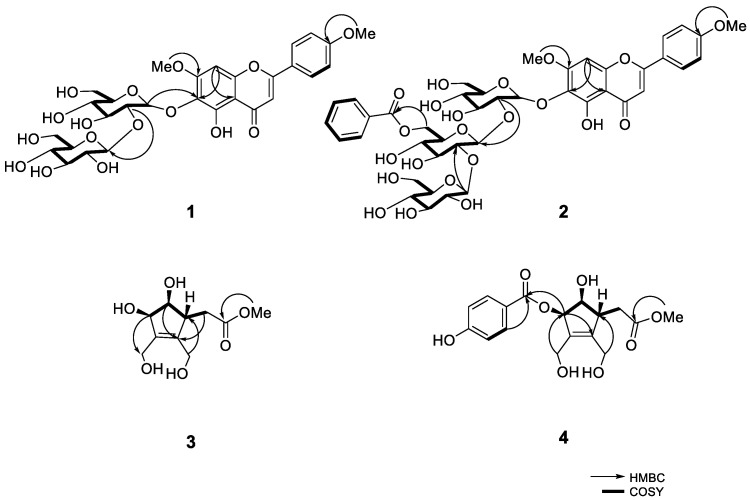
The key HMBC and COSY correlations of **1**−**4**.

Compound **1** was isolated as a yellow amorphous powder. Its molecular formula was determined as C_29_H_34_O_16_ based on HR-ESI-MS pseudo-ion at *m/z* 639.1946 [M + H]^+^ (calcd. for C_29_H_35_O_16_, 639.1920). The ^13^C-NMR and DEPT spectra of **1** showed the presence of 29 carbons, including nine non-protonated carbons (one carbonyl, five oxygenated), 16 methines (ten oxygenated), two oxygenated methylenes, and two methoxy carbons (Table 1). The HMBC correlations between proton signals of two methoxys at *δ*_H_ 3.82 and 3.86 (each 3H, s) and C-4’ (*δ*_C_ 162.4) and C-7 (*δ*_C_ 158.3) suggested their locations at C-4’ and C-7, respectively (Figure 2). Besides, the location of a sophoroside was also confirmed by HMBC correlation between H-1’’ (*δ*_H_ 5.29) and C-6 (*δ*_C_ 127.9). Compound **1** was identified as 5,6-dihydroxy-7,4’-dimethoxyflavone-6-*O*-sophoroside, which was previously identified from *C. ovata* [9]. Although its ^1^H-NMR data were reported, ^13^C-NMR data were not fully assigned. Therefore, we herein report fully assigned ^13^C-NMR data. The UV, IR, CD, NMR (^1^H, ^13^C, HSQC and HMBC) and HR-ESI-MS spectrum of compound **1** are shown in Supplementary Figures S1–S8. 

Compound **2** was isolated as a yellow amorphous powder. Its molecular formula was determined as C_42_H_48_O_22_ on the basis of HR-ESI-MS pseudo-ion at *m/z* 905.2669 [M + H]^+^ (calcd. for C_42_H_49_O_22_, 905.2710). The ^1^H-NMR spectrum of **2** showed the signals of two methoxy protons at *δ*_H_ 3.83 and 3.88 (each 3H, s), three anomeric protons at *δ*_H_ 4.71 (d, *J* = 7.7 Hz), 4.74 (d, *J* = 7.6 Hz), and 5.28 (d, *J* = 7.0 Hz), and two olefinic protons at *δ*_H_ 6.53 (s) and 6.43 (s). In addition, proton signals of A_2_B_2_-type aromatic protons at *δ*_H_ 7.08 (d, *J* = 8.4 Hz) and 7.90 (d, *J* = 8.4 Hz), and a benzoyl group at *δ*_H_ 7.13 (t, *J* = 7.4 Hz), 7.29 (t, *J* = 7.4 Hz), and 7.66 (2H, m) were shown. The ^13^C-NMR and HSQC spectra of **2** showed the presence of 42 carbons, including eleven non-protonated carbons (two carbonyls), 26 methines (fifteen oxygenated), three oxygenated methylenes, and two methoxy carbons (Table 1). The analysis NMR data of **2** indicated that the structure of **2** was similar to those of 5,6-dihydroxy-7,4′-dimethoxyflavone-6-*O*-sophoroside [9], except for the addition of a 6-*O*-benzoate glucopyranoside. The position of a sugar moiety at C-6 was suggested by HMBC correlations from glc H-1’’ (*δ*_H_ 5.28) to C-6 (*δ*_C_ 128.0). The sugar moiety was identified by comparing NMR data with those of sugar moiety in 6-hydroxyluteolin 7-*O*-[6‴-benzoyl-β-d-glucopyranosyl-(1→2)]-β-d-glucopyranoside (aphyllanthoside) [18]. The terminal sugar connectivity was confirmed by HMBC correlation between H-2’’’ (*δ*_H_ 3.56) and C-1’’’’ (*δ*_C_ 104.4), which proved a 1→2 interglycosidic linkage by the downfield shift for C-2’’’ (*δ*_C_ 83.1) of the inner glucose. The position of benzoyl group was verified by the 2D NMR and the MS/MS spectrum. The esterification site of the benzoic acid was found to be C-6’’’ of the terminal glucose, on the basis of the de-shielding of H-6’’’ (*δ*_H_ 4.33) and C-6’’’ (*δ*_C_ 64.0) as well as the cross-peak at C-7’’’’’ (*δ*_C_ 166.3) in the HMBC spectrum. In addition, it was further confirmed by MS/MS spectrum of compound **2** to elucidate the location of the benzoyl group. Its two fragment ions, Y1 and Y2, generated by the cleavage of sugar moieties from compound **2** (Y1 [M + H]^+^ and Y2 [M + Na – H_2_O]^+^) using LC-QTOF-MS revealed that the benzoyl group is located at the second sugar. The positions of methoxy groups were verified by the HMBC correlation between protons of methoxy groups (*δ*_H_ 3.83 and 3.88) and C-7 and C-4’ (*δ*_C_ 158.4 and 162.9), concluding that the methoxy groups are located at C-7 and C-4’, respectively (Figure 2). Thus, the structure of **2** was elucidated to be 5,6-dihydroxy-7,4’-dimethoxyflavone-6-*O*-[6’’’-benzoyl-β-d-glucopyranosyl-(1→2)-β-d-glucopyranosyl-(1→2)]-β-d-glucopyranoside. The UV, IR, CD, NMR (^1^H, ^13^C, HSQC and HMBC), HR-ESI-MS and MS/MS spectrum of compound **2** are shown in Supplementary Figures S9–S17.

**Figure 3:**
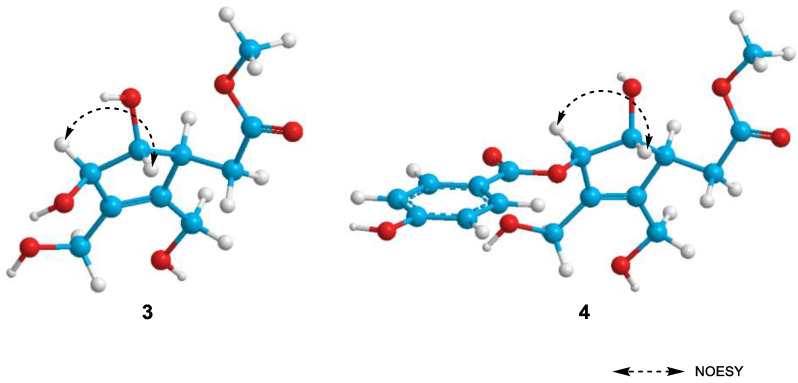
The key NOESY correlation of compounds **3** and **4**.

**Figure 4:**
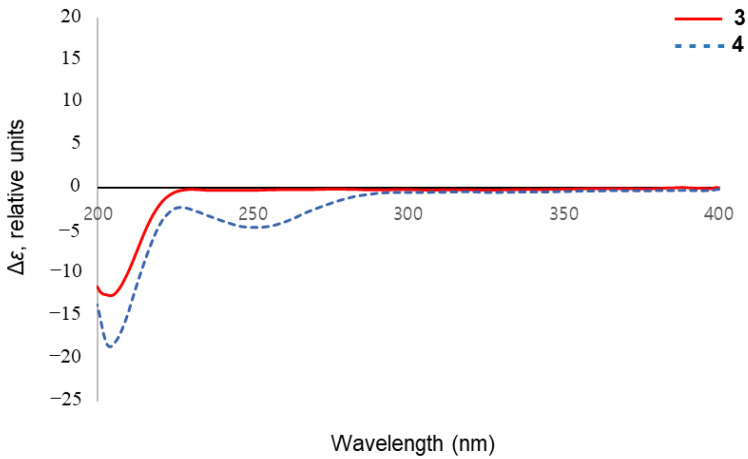
CD spectra of compounds **3** and **4**.

Compound **3** was isolated as a brown oil. Its molecular formula was determined as C_10_H_16_O_6_ based on HR-ESI-MS pseudo-ion at *m/z* 233.0981 [M + H]^+^ (calcd. for C_10_H_17_O_6_, 233.1020). The NMR spectra were similar to those of (7*R*)-hydroxyeucommic acid isolated from the fruits *C*. *ovata* [10], except for the replacement of a hydroxyl group into a methoxy group at C-3. The ^1^H-NMR spectrum of **3** showed the signals of one methoxy proton at *δ*_H_ 3.75 (3H, s). The methoxy group showed an HMBC correlation with ester carbonyl carbon (*δ*_C_ 174.9), which was also correlated with methylene protons at H-4 (*δ*_H_ 2.48) (Figure 2). The stereochemistry of **3** was clarified based on the NOESY and the CD spectra. The NOE interaction was observed between H-6 (*δ*_H_ 3.95) and H-7 (*δ*_H_ 4.56), but no correlation was shown between H-5 (*δ*_H_ 3.17) and H-6 (*δ*_H_ 3.95) (Figure 3). The absolute configuration was determined by the application of the inverse Octant rule for allylic oxygen substituent, which generally dominated the onset or appearance of the lower wavelength Cotton effect [19]. In addition, iridoids isolated from *Catalpa* genus such as (7*R*)-hydroxyeucommic acid *n*-butyl ester [Δ*ε* −0.67 (204.4 nm)] [10] exhibited *R* configuration. As a biogenetic derivative, compound **3** is supposed to have an *R* configuration at C-7. It was further confirmed by CD spectrum with a negative Cotton effect at 204.0 nm (Δ*ε* −12.65) (Figure 4). The structure of **3** is, therefore, determined as (7*R*)-3-methoxy-hydroxyeucommic acid. The IR, NMR (^1^H, ^13^C, HSQC and HMBC) and HR-ESI-MS spectrum of compound **3** are shown in Supplementary Figures S18–S23.

Compound **4** was also isolated as a brown oil. Its molecular formula was confirmed as C_17_H_20_O_8_ by HR-ESI-MS ion at *m/z* 353.1207 [M + H]^+^ (calcd. for C_17_H_21_O_8_, 353.1231). The ^1^H- and ^13^C-NMR spectrum of **4** also exhibited (7*R*)-hydroxyeucommic acid analog as those of compound **3** (Table 2). Instead of a hydroxyl group at C-7 in compound **3**, compound **4** consisted of a *p*-hydroxybenzoic acid. The HMBC spectrum gave a three-bonded correlation between C-11 (*δ*_C_ 168.1) and H-7 (*δ*_H_ 5.87) (Figure 2). The stereochemistry of **4** was also determined by NOESY and the CD spectra, as with compound **3**. The NOE interaction was observed between H-6 (*δ*_H_ 4.17) and H-7 (*δ*_H_ 5.87), but not between H-5 (*δ*_H_ 3.24) and H-6 (*δ*_H_ 4.17) (Figure 3). The CD spectrum of **4** also showed a negative Cotton effect at 204.0 nm (Δ*ε* −18.57), suggesting its *R*-configuration at C-7 (Figure 4). Thus, the structure of **4** was elucidated to be (7*R*)-3-methoxy-(7-*O*-*p*-hydroxybenzoyl)eucommic acid. The UV, IR, NMR (^1^H, ^13^C, HSQC and HMBC) and HR-ESI-MS of compound **4** are shown in Supplementary Figures S24–S30.

### 2.2. α-Glucosidase Inhibitory Activity

In spite of the introduction of various anti-DM medicines such as DPP-4 inhibitors, SGLT-2 inhibitors, or GLP-1 analogues, α-glucosidase inhibitors are still prevalently used to establish glycemic control over postprandial hyperglycemia. They can retard the liberation of glucose from carbohydrates and delay glucose absorptions from the gut. To find anti-DM phytochemicals, all of the isolated compounds were evaluated for α-glucosidase inhibitory activity using enzyme assay at the concentration of 20 μM (Table 3). The previous biological evaluation suggested that anthocyanidin, isoflavone, flavonol, and secoiridoid glucosides exhibit potent inhibitory effects on α-glucosidase activity [20,21]. In accordance with previous biological data, compounds **2** (flavone glycoside), **3**, and **9** (both iridoids) demonstrated the most potent inhibitory activity, which is comparable to that of a well-known α-glucosidase inhibitor, acarbose. Several previous researchers have reported that flavones, such as isoquercitrin and isovitexin [22], as well as the iridoid, such as loniceranan B and swreoside [20], exerted significant α-glucosidase inhibitory activities. Thus, compounds **2**, **3**, and **9** could be potential natural resources as anti-DM phytochemicals.

### 2.3. Virtual Screening of α-Glucosidase Inhibitors

**Figure 5:**
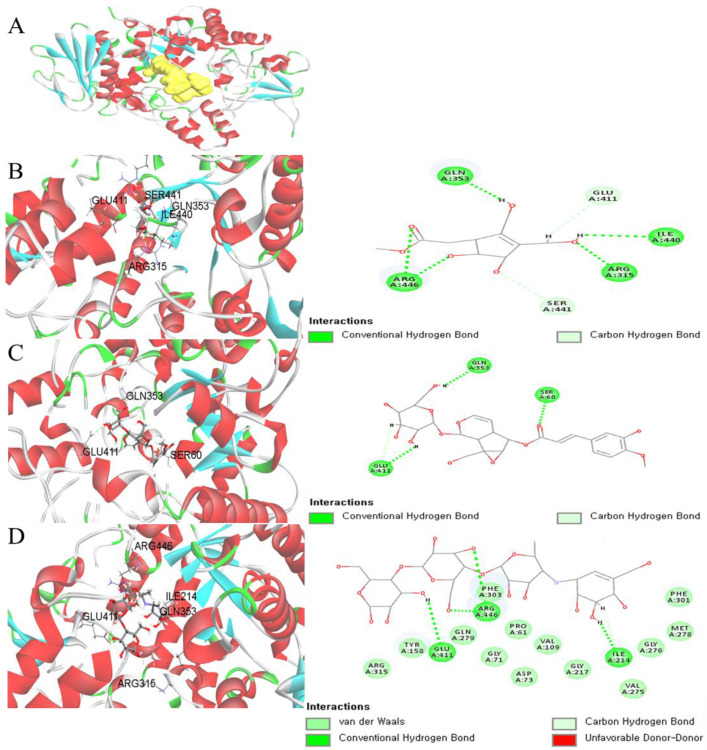
Binding mode of **3**, **9**, and acarbose in the active site of a-glucosidase (**A**). Receptor–ligand interaction of compounds **3** (**B**), **9** (**C**), and acarbose (**D**) on 2D and 3D diagram.

Virtual screening (VS) could be useful in searching for a novel lead compound that is appropriate for predictive new drug discovery studies. Compared with other de novo design methods, virtual screening suggests a proper understanding of the important structural and physicochemical features. To identify the putative binding conformation of bioactive compounds, the structure-based VS was performed against the α-glucosidase protein. The crystal structure of α-glucosidase complexed with inhibitor was obtained from the Protein Data Bank (PDB, http://www.rcsb.org/pdb) under code (PDB 3A4A). Compounds **3** and **9** were well-docked into the catalytic site of the α-glucosidase (Figure 5) with the values of CDOCKER energy of −17.98 and −22.64 kcal·mol^−1^, respectively. The key feature showed that **3** was docked into the active site via conventional hydrogen bonds with residues Arg 315, Arg 446, Gln 353, and Ile 440, and carbon–hydrogen bond with residues Glu 411 and Ser 441. Compound **9** formed conventional hydrogen bonding with residues Gln 353, Glu 411, and Ser 60.

### 2.4. Glucose-Stimulated Insulin Secretion (GSIS) Effect

**Figure 6:**
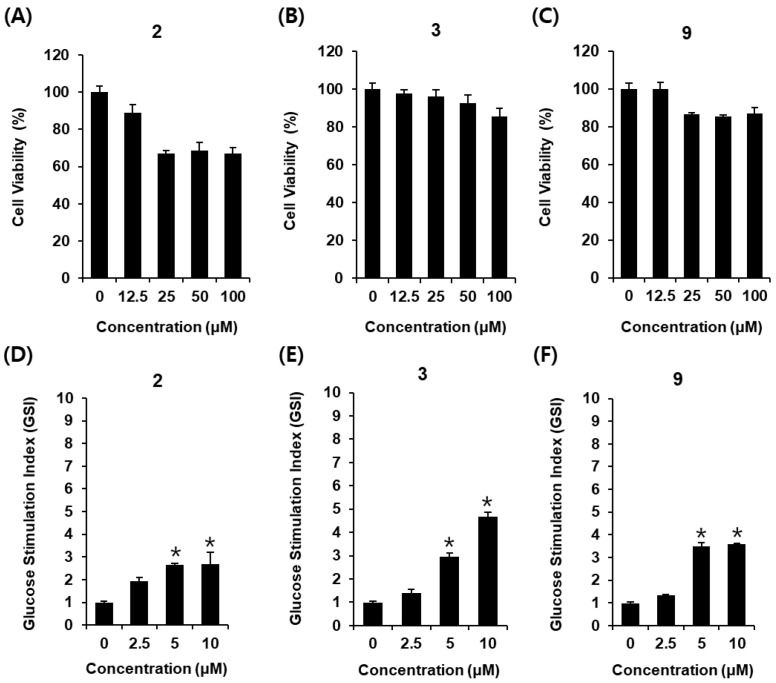
Effect of compounds **2**, **3**, and **9** on glucose-stimulated insulin secretion in INS-1 cells. Effect of (**A**) compounds **2**, (**B**) **3**, and (**C**) **9** on the viability of INS-1 cells following 24 h of treatment, compared with that of the control (0 μM), as determined by the MTT assay. Effect of (**D**) compounds **2**, (**E**) **3**, and (**F**) **9** on glucose-stimulated insulin secretion in INS-1 cells following 1 h of treatment, compared with that of the control (0 μM), as determined by the GSIS assay (*n* = 3 independent experiments, * *p* < 0.05 compared to the control (0 μM), Kruskal–Wallis non-parametric test). The data are presented as the mean ± SEM.

Dysfunction and mass loss of pancreatic β cells are known to be risk factors for developing type 2 diabetes. Impairment of GSIS is mainly attributed to the initial dysfunction of pancreatic β cells. Therefore, amelioration of GSIS might be a strategy for the discovery of a potential antidiabetic agent. In the present study, compounds **2**, **3**, and **9**, which exerted significant α-glucosidase inhibitory activities, were determined to increase GSIS in INS-1 cells. Since compounds **2**, **3**, and **9** were not toxic in less than 12.5 μM, their concentrations were used in the insulin secretion assay (Figure 6A–C). As shown in Figure 6D–F, compounds **2**, **3**, and **9** led to an increase in GSI in a dose-dependent manner. The GSI levels were 1.96 ± 0.16, 2.71 ± 0.05, and 2.73 ± 0.51 for compound **2** at 2.5, 5, and 10 μM, respectively (Figure 6D). The GSI levels were 1.37 ± 0.01, 3.56 ± 0.14, and 3.66 ± 0.03 for compound **3** at 2.5, 5, and 10 μM, respectively (Figure 6E). The GSI levels were 1.43 ± 0.16, 3.02 ± 0.13, and 4.75 ± 0.21 for compound **9** at 2.5, 5, and 10 μM, respectively (Figure 6F). Compounds **2**, **3**, and **9** stimulated insulin secretion in INS-1 cells without inducing cytotoxicity. In the amelioration of GSIS in INS-1 cells, compounds **3** and **9** (both iridoids) were more effective than compound **2** (flavone glycoside). The insulin secretion effect of compound **3** was the best and increased in a concentration-dependent manner. It has been shown that lyonofolin B, an iridoid isolated from *lyonia ovalifolia*, potentiated a GSIS from mice pancreatic islets in the male BALB/c mice [23]. Moreover, another study has indicated that rutin, a flavonoid glycoside, increases GSIS from pancreatic islets in the male Wistar rats [24]. However, its underlying mechanism has yet to be revealed. A further study revealed the underlying mechanism of compounds **2**, **3**, and **9** on amelioration of GSIS using the Western blot assay.

### 2.5. Protein Expression of PPARγ, P-IRS-2, IRS-2 (Ser731), P-PI3K, PI3K, P-Akt (Ser473), Akt, and PDX-1

**Figure 7:**
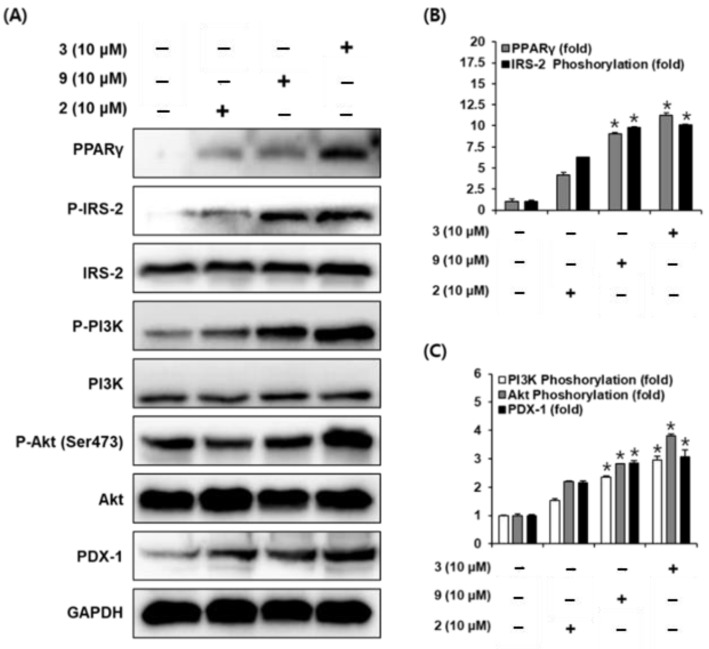
Effect of compounds **2**, **3**, and **9** on the protein expression levels of peroxisome proliferator-activated receptor-γ (PPAR-γ), P-insulin receptor substrate-2 (IRS-2) (Ser731), IRS-2, P-phosphatidylinositol 3-kinase (PI3K), PI3K, P-Akt (Ser473), Akt, and pancreatic and duodenal homeobox-1 (PDX-1) in INS-1 cells. (**A**) Protein expression levels of PPAR-γ, P-IRS-2 (Ser731), IRS-2, P-PI3K, PI3K, P-Akt (Ser473), Akt, PDX-1, and glyceraldehyde 3-phosphate dehydrogenase (GAPDH) in INS-1 cells treated or untreated with 10 μM compounds **2**, **3**, and **9** for 24 h. (**B**,**C**) Each bar graph presents the densitometric quantification of Western blot bands. * *p* < 0.05 compared to the control (0 μM), Kruskal–Wallis non-parametric test). The data are presented as the mean ± SEM.

To evaluate the role of peroxisome proliferator-activated receptor γ (PPAR-γ), insulin receptor substrate-2 (IRS-2), phosphatidylinositol 3-kinase (PI3K), Akt, and pancreatic duodenal homeobox-1 (PDX-1) in the effect of compounds **2**, **3**, and **9** on GSIS, we measured these protein levels in pancreatic β-cells and demonstrated that the protein expression levels of PPAR-γ, P-IRS-2 (Ser731), P-PI3K, P-Akt (Ser473), and PDX-1 were increased by treatment with compounds **2**, **3**, and **9** at 10 μM compared to untreated controls. As reported, PPARγ has been shown to regulate the expression of genes involved in insulin secretion in pancreatic β cells [25]. However, in order to avoid the potential side effects of synthetic antidiabetic compounds such as thiazolidinediones and troglitazone, the PPAR-γ agonists, finding antidiabetic compounds from natural products is necessitated. Another role of PPAR-γ is known to regulate the PDX-1 gene promoter in pancreatic β cells [26,27]. Others have reported that troglitazone increases the expression of PDX-1 in INS-1 cells [26]. This prompted us to study the effect of compounds **2**, **3**, and **9** on the expression of PPAR-γ and PDX-1 in INS-1 cells. Thus, the expression of PPAR-γ and PDX-1 was evaluated with Western blot. As shown in Figure 7, treatment with compounds **2**, **3**, and **9** increased the expression of PPAR-γ and PDX-1. Subsequently, we assessed whether treatment with compounds **2**, **3**, and **9** increases the serine phosphorylation of IRS-2 (Ser 731), phosphorylation of PI3K, and serine phosphorylation of Akt (Ser473). It is well known that the IRS-2 signaling pathway is essential to the function of pancreatic β cells. In addition, the loss of IRS-2 expression in mice is linked to development of type 2 diabetes due to insufficiency of pancreatic β cells [28]. In this signaling pathway, Akt and its downstream protein, PI3K, can be activated through phosphorylation of IRS-2 [29]. It has also been reported that upregulation of the PI3K/Akt signaling pathway promotes proliferation of pancreatic β cells [30]. As shown in Figure 7, treatment with compounds **2**, **3**, and **9** increased the expression of IRS-2, PI3K, and Akt. Taken together, these results suggested that compounds **2**, **3**, and **9** not only upregulate the expression of PPAR-γ and PDX-1, but also upregulate the phosphorylation of IRS-2, PI3K, and Akt in INS-1 cells. These results enhanced the understanding of the underlying mechanism of compounds **2**, **3**, and **9** on amelioration of GSIS. However, further study on the mode of entry of compounds **2**, **3**, and **9** into the pancreatic β cells and its effect in animal models for diabetes should be evaluated.

## 3. Materials and Methods

### 3.1. General Experimental Procedures

Chemical shifts are reported in parts per million from TMS. All NMR spectra were recorded on an Agilent 400-MR-NMR spectrometer (Santa Clara, CA, USA) operated at 400 and 100 MHz for hydrogen and carbon, respectively. Data processing was carried out with the MestReNova ver.6.0.2 program. HR-ESI-MS spectra were obtained using an AGILENT 6550 iFunnel Q-TOF LC/MS system (Santa Clara, CA, USA). Optical rotations were determined on a Jasco DIP-370 automatic polarimeter. Circular dichroism spectrums were determined on a Chirascan™ CD spectrometer. Preparative HPLC was carried out using an AGILENT 1200 HPLC system. Column chromatography was performed on silica gel (Kieselgel 60, 70-230 mesh and 230-400 mesh, Merck, Darmstadt, Germany) or YMC RP-18 resins (30–50 μm, Fuji Silysia Chemical Ltd. Aichi, Japan). For thin-layer chromatography (TLC), a pre-coated silica-gel 60 F254 (0.25 mm, Merck) and RP-18 F254S plates (0.25 mm, Merck) were used.

### 3.2. Plant Material

The fruits of *C. bignonioides* were collected from Arboretum of Seoul National University in Suwon, Korea, in 2017 and authenticated by Dr. Rack-Seon Seong, a director of the Center of Natural Resources Research, Jeonnam Bioindustry Foundation. A voucher specimen (CB201709) is deposited at the Herbarium of College of Pharmacy, Yonsei Institute of Pharmaceutical Sciences, Yonsei University, Incheon, Korea.

### 3.3. Isolation of Compounds ***1**–**15***

Dried fruits of *Catalpa bignonioides* (1.3 kg) were extracted with MeOH (5 L × 3 times) under sonication at 30 °C for 4 h to yield an extract (91.0 g), which was then suspended in H_2_O and successively partitioned using CHCl_3_ and EtOAc to obtain CHCl_3_ (CB1, 16.0 g), EtOAc (CB2, 2.5 g), and H_2_O (CB3, 71.0 g) extracts after removal of the solvents in vacuo.

The CHCl_3_ fraction (CB1, 16.0 g) was subjected to a silica gel CC, and eluting with a gradient of hexane:acetone (40:1 → 2.5:1, *v*/*v*) and CHCl_3_:MeOH (20:1 → 2.5:1, *v*/*v*) gave nine sub-fractions: CB1A (3.0 g), CB1B (2.4 g), CB1C (1.0 g), CB1D (1.5 g), CB1E (1.0 g), CB1F (1.2 g), CB1G (0.8 g), CB1H (1.0 g), and CB1I (0.5 g). The CB1F fraction was applied to an YMC RP-18 column, which when eluted with MeOH:H_2_O (1.3:1, *v*/*v*) gave four smaller fractions: CB1F1 (58.2 mg), CB1F2 (41.5 mg), CB1F3 (18.4 mg), and CB1F4 (16.5 mg), respectively. The CB1F1 fraction was subjected to HPLC, using J’sphere ODS H-80 250 × 20 mm column, eluted with MeCN:H_2_O (28:72), and a flow rate of 3 mL/min to yield **10** (6.8 mg) and **11** (7.1 mg). The CB1F2 fraction was subjected to the same HPLC conditions, except that the eluding solvent was MeCN:H_2_O (40:60), to afford **12** (8.1 mg).

The H_2_O fraction (CB3, 71.0 g) was chromatographed on a Diaion HP-20 column eluting with H_2_O containing an increasing concentration of MeOH (25%, 50%, and 100%) to obtain three sub-fractions: CB3A (10.0 g), CB3B (13.0 g), and CB3C (6.0 g). The CB3B fraction was subjected to a silica gel CC and eluting with a gradient of CHCl_3_:MeOH (10:1 → 2.5:1, *v*/*v*) gave three sub-fractions: CB3B1 (2.0 g), CB3B2 (2.7 g), and CB3B3 (2.0 g). The CB3B1 fraction was applied to a silica gel column, which when eluted with CHCl_3_:MeOH:H_2_O (5:1:0.1, *v*/*v*) gave six smaller fractions: CB3B11 (31.0 mg), CB3B12 (96.0 mg), CB3B13 (82.0 mg), CB3B14 (150.4 mg), CB3B15 (66.7 mg), and CB3B16 (213.8 mg), respectively. The CB3B14 fraction was applied to HPLC purification as above to yield **4** (6.8 mg) and **5** (26.2 mg). The CB3B16 fraction was subjected to the same HPLC conditions, except that the eluding solvent was MeCN:H_2_O (23:77), to afford **3** (140.0 mg). The CB3C fraction was subjected to a silica gel CC and eluting with a gradient of CHCl_3_:MeOH (10:1 → 2.5:1, *v*/*v*) gave three sub-fractions: CB3C1 (0.4 g), CB3C2 (1.5 g), and CB3C3 (1.0 g). The CB3C1 fraction was applied to an YMC RP-18 column, which when eluted with MeOH:H_2_O (1:1, *v*/*v*) gave three smaller fractions: CB3C11 (0.2 g), CB3C12 (55.5 mg), and CB3C13 (14.0 mg), respectively. The CB3C11 fraction was subjected to HPLC, using J’sphere ODS H-80 250 × 20 mm column, eluted with MeCN:H_2_O (18:82), and a flow rate of 3 mL/min to yield **13** (55.9 mg), **14** (7.3 mg), and **15** (14.3 mg). The CB3C2 fraction was applied to an YMC RP-18 column, which when eluted with MeOH:H_2_O (1:1, *v*/*v*) gave three smaller fractions: CB3C21 (0.2 g), CB3C22 (0.6 g), and CB3C23 (0.2 g), respectively. The CB3C21 fraction was subjected to HPLC, using J’sphere ODS H-80 250 × 20 mm column, eluted with MeCN:H_2_O (30:70), and a flow rate of 3 mL/min to yield **6** (35.5 mg), whereas the CB3C23 fraction gave **7** (30.1 mg), **8** (18.5 mg), and **9** (6.3 mg). The CB3C3 fraction was applied to an YMC RP-18 column, which when eluted with MeOH:H_2_O (1.4:1, *v*/*v*) gave four smaller fractions: CB3C31 (42.8 mg), CB3C32 (0.1 g), CB3C33 (30.8 mg), and CB3C34 (18.6 g), respectively. The CB3C32 fraction was subjected to HPLC, using J’sphere ODS H-80 250 × 20 mm column, eluted with MeCN:H_2_O (25:75), and a flow rate of 3 mL/min to yield **1** (11.3 mg). The CB3C34 fraction was subjected to the same HPLC conditions, except that the eluting solvent was MeCN:H_2_O (23:77), to afford **2** (7.1 mg).

*5,6-dihydroxy-7,4’-dimethoxyflavone-6-O-sophoroside* (**1**). Yellow amorphous powder; C_29_H_34_O_16_, HR-ESI-MS *m/z*: 639.1946 [M + H]^+^ (calcd. for C_29_H_35_O_16_, 639.1920); UV (MeOH) λ_max_ (log ε) 335 (0.40) nm, 275 (0.28); IR (KBr)*ν*_max_ 3294, 2842, 1639, 1449, 1410, 1113, 1013 cm^−1^; ^1^H- (DMSO, 400 MHz) and ^13^C-NMR (DMSO, 100 MHz) data, see Table 1.

*5,6-dihydroxy-7,4′-dimethoxyflavone-6-O-[6’’’-benzoyl-β-d-glucopyranosyl-(1→2)-β-d-glucopyranosyl-(1→2)]-β-d-glucopyranoside* (**2**). Yellow amorphous powder; C_42_H_48_O_22_, HR-ESI-MS *m/z*: 905.2669 [M + H]^+^ (calcd. for C_42_H_49_O_22_, 905.2710); UV (MeOH) λ_max_ (log ε) 331 (0.40), 279 (0.33), 217 (0.60) nm; IR (KBr)*ν*_max_ 3311, 2944, 2831, 1655, 1449, 1116, 1022 cm^−1^; ^1^H- (CD_3_OD, 400 MHz) and ^13^C-NMR (CD_3_OD, 100 MHz) data, see Table 1.

*(7R)-3-methoxy-hydroxyeucommic acid* (**3**). Brown oil; CD (c = 2 × 10^−4^, MeOH) Δ*ε* (nm) –12.65 (204); C_10_H_16_O_6_, HR-ESI-MS *m/z*: 233.0981 [M + H]^+^ (calcd. for C_10_H_17_O_6_, 233.1020); IR (KBr)*ν*_max_ 3310, 2944, 2832, 1719, 1439, 1411, 1163, 1095, 1021 cm^−1^; ^1^H- (CD_3_OD, 400 MHz) and ^13^C-NMR (CD_3_OD, 100 MHz) data, see Table 2.

*(7R)-3-methoxy-(7-O-p-hydroxybenzoyl)eucommic acid* (**4**). Brown oil; CD (c = 2 × 10^−4^, MeOH) Δ*ε* (nm) –18.57 (204); C_17_H_20_O_8_, HR-ESI-MS *m/z*: 353.1207 [M + H]^+^ (calcd. for C_17_H_21_O_8_, 353.1231); UV (MeOH) λ_max_ (log ε) 257 (0.52) nm; IR (KBr)*ν*_max_ 3308, 2944, 2832, 1716, 1448, 1418, 1273, 1114, 1022 cm^−1^; ^1^H- (CD_3_OD, 400 MHz) and ^13^C-NMR (CD_3_OD, 100 MHz) data, see Table 2.

### 3.4. Assay of α-Glucosidase Activity

α-Glucosidase was assayed using Sigma-Aldrich commercial kits (Art. No. MAK123, St. Louis, MO, USA) according to the manufacturer’s instructions. Briefly, 20 μL of isolated compounds (20 μM) was mixed with 200 μL α-glucosidase enzyme solution and incubated at 37 °C for 20 min, and absorbance was measured at 405 nm in a plate reader (Tecan, Crailsheim, Germany). Acarbose was used as a positive control. The α-glucosidase activity was calculated by the following formula:

(1) $$\alpha \mathrm{-glucosidase}\;\mathrm{activity}\;\left(\mathrm{units}/L\right)=\frac{{\left(A_{405}\right)}_{\mathrm{final}-{\left(A_{405}\right)}_{\mathrm{initial}}}}{{\left(A_{405}\right)}_{\mathrm{calibrator}-{\left(A_{405}\right)}_{\mathrm{water}}}}\times 250\;\mathrm{units}/L$$

### 3.5. Structured-Based Virtual Screening

Structure-based virtual screening was performed using CDOCKER docking protocol in Discovery studio (BIOVIA/Accelrys Inc. San Diego, CA, USA). The 3A4A of α-glucosidase was downloaded from RCSB Protein Data Bank (www.rcsb.org). To perform docking in CDOCKER, the following docking parameters were used: grid center (X: 20.62, Y: −2.54. Z: 18.25), grid size (14.2 Å), grid spacing (0.5 Å). The most potent α-glucosidase inhibitory compounds, **2**, **3**, and **9**, and positive control, acarbose, were used as a ligand. The hydrogen atoms were added to ligands. The 3D structure of ligands was generated through molecular dynamics, and the initial poses were generated by various rotations/translations of structures. Both ligand and receptor docking were performed using Chemistry at Harvard Macromolecular Mechanics (CHARMm) force field docking algorithm (Runs 10). For defined electrostatic or van der Waals interaction, a +1 charge probe was used for electrostatic interaction, and probe radii (0.65 through 2.55 Å) was used for van der Waals interaction. The known active site of α-glucosidase was selected as the binding site and −CDOCKER energy level was used for the result of interactions between the ligands and the receptor.

### 3.6. Cell Culture

Rat pancreatic β-cell line INS-1 (Biohermes, Shanghai, China) were cultivated in an RPMI-1640 medium (Cellgro, Manassas, VA, USA) supplemented with 10% fetal bovine serum (FBS), 1% penicillin/streptomycin (Invitrogen Co., Grand Island, NY, USA), 11 mM d-glucose, 2 mM l-glutamine, 10 mM HEPES, 0.05 mM 2-mercaptoethanol, and 1 mM sodium pyruvate at 37 °C in a CO_2_ incubator (5% CO_2_ in air).

### 3.7. Cell Viability Assay

To determine the non-toxic dose ranges of compounds **2**, **3**, and **9**, cell viability was measured using the Ez-Cytox cell viability detection kit (Daeil Lab Service Co., Seoul, Korea). INS-1 cells were seeded in each well of 96-well plates for 24 h and treated with compounds **2**, **3**, and **9** for 24 h, followed by 2 h incubation at 37 °C. The absorbance values at 450 nm of each well were recorded using a PowerWave XS microplate reader (Bio-Tek Instruments, Winooski, VT, USA).

### 3.8. GSIS Assay

GSIS assay was performed in INS-1 cells after treatment with compounds **2**, **3**, and **9**. INS-1 cells were seeded in each well of 12-well plates for 24 h and washed with Krebs-Ringer bicarbonate HEPES buffer (KRBB, 4.8 mM KCl, 129 mM NaCl, 1.2 mM KH_2_PO_4_, 1.2 mM MgSO_4_, 2.5 mM CaCl_2_, 10 mM HEPES, 5 mM NaHCO3, and 0.1% bovine serum albumin (BSA), pH 7.4) and 2.8 mM glucose, twice.

After starvation in fresh KRBB for 2 h, the cells were treated with compounds **2**, **3**, and **9** for 2 h, following which they were stimulated with 2.8 and 16.7 mM glucose, respectively. After incubation in 2.8 and 16.7 mM glucose respectively, for 1 h, GSIS was measured with supernatants from each well using a rat insulin ELISA kit (Gentaur, Shibayagi Co. Ltd., Gunma, Shibukaw, Japan) according to the manufacturer’s instructions. The glucose stimulation index (GSI) was calculated by dividing insulin concentration secreted during exposure to 2.8 mM glucose by insulin concentration secreted during exposure to 16.7 mM glucose.

### 3.9. Western Blot Analysis

The expression of proteins was measured by Western blot analysis. After treatment with compounds **2**, **3**, and **9** for 24 h, an equal amount of protein lysate (20 μg per lane) from INS-1 cells was added. After 10% sodium dodecyl sulfate-polyacrylamide gel electrophoresis, the proteins were transferred to polyvinylidene difluoride (PVDF) membranes. PVDF membranes were probed with primary antibodies against PPARγ, P-IRS-2 (Ser731), IRS-2, P-PI3K, PI3K, P-Akt (Ser473), Akt, PDX-1, and glyceraldehyde 3-phosphate dehydrogenase (GAPDH) (Cell Signaling, Danvers, MA, USA) for 1 h at 4 °C, followed by horseradish peroxidase (HRP)-conjugated anti-rabbit secondary antibodies (Cell Signaling, Boston, MA, USA) for 1 h at 4 °C. The probed blots after treatment with an enhanced chemiluminescence reagent (GE Healthcare UK Limited, Buckinghamshire, UK) were visualized using a chemiluminescence system (FUSION Solo, PEQLAB Biotechnologie GmbH, Erlangen, Germany).

### 3.10. Statistical Analysis

Statistical significance was determined using one-way analysis of variance (ANOVA) and multiple comparisons with a Bonferroni correction. *p*-values of less than 0.05 indicated statistical significance. All analyses were performed using SPSS Statistics ver. 19.0 (SPSS Inc., Chicago, IL, USA).

## 4. Conclusions

The detailed phytochemical study of fruits of *C. bignonioides* resulted in the isolation of three new compounds (**2**–**4**) along with twelve previously reported compounds (**1** and **5**–**15**). Among the known compounds, 5,6-dihydroxy-7,4’-dimethoxyflavone-6-*O*-sophoroside (**1**) was reported with the fully assigned ^13^C-NMR data for the first time in the present investigation. To identify natural antidiabetic compounds, isolated compounds were tested for α-glucosidase inhibitory activity and the GSIS effect. Compounds **2**, **3**, and **9** exerted significant α-glucosidase inhibitory activities and GSIS effect. Moreover, the GSIS effects of these three active compounds were supported by the increased expressions of PPAR-γ, IRS-2, PI3K, Akt, and PDX-1. These results demonstrated that phytochemicals isolated from *C. bignonioides* could be an alternative option for treating diabetes, contributing to glucose homeostasis.

## Figures and Tables

**Table 1:** NMR spectroscopic data for compounds **1** and **2**.

	1			2	
Pos.	*δ*_C_^a,b^	*δ*_H_^a,d^ (*J* in Hz)	Pos.	*δ*_C_^a,b^	*δ*_H_^a,c^ (*J* in Hz)
1			1		-
2	163.5	-	2	164.1	-
3	103.4	6.90 (s)	3	102.9	6.53 (s)
4	182.2	-	4	182.5	-
5	151.0	-	5	152.8	-
6	127.9	-	6	128.0	-
7	158.3	-	7	158.4	-
8	91.9	6.92 (s)	8	90.9	6.43 (s)
9	152.5	-	9	153.1	-
10	104.9	-	10	105.0	-
1’	122.8	-	1’	123.0	-
2’, 6’	128.4	8.04 (d, 8.9)	2’, 6’	127.8	7.90 (d, 8.4)
3’, 5’	114.6	7.09 (d, 9.0)	3’, 5’	114.2	7.08 (d, 8.4)
4’	162.4	-	4’	162.9	-
1’’	99.6	5.30 (d, 6.5)	1’’	100.4	5.28 (d, 7.0)
2’’	81.3	3.63 *	2’’	85.0	3.62 *
3’’	76.3	3.45 *	3’’	75.7	3.65 *
4’’	69.9	3.05 *	4’’	68.8	3.50 *
5’’	77.0	3.15 *	5’’	76.1	3.20 *
6’’	60.9	3.54 *	6’’	60.9	3.67 *
1’’’	102.9	4.57 (d, 7.9)	1’’’	102.9	4.74 (d, 7.6)
2’’’	74.1	2.98 *	2’’’	83.1	3.56 *
3’’’	76.4	3.12 *	3’’’	76.6	3.64 *
4’’’	69.2	3.33 *	4’’’	69.6	3.34 *
5’’’	76.9	3.05 *	5’’’	76.9	3.20 *
6’’’	60.8	3.40 *	6’’’	64.0	4.33 (d, 3.8)
			1’’’’	104.4	4.71 (d, 7.7)
			2’’’’	74.4	3.28 *
			3’’’’	76.3	3.37 *
			4’’’’	69.9	3.37 *
			5’’’’	77.1	3.35 *
			6’’’’	60.9	3.62 *
			1’’’’’	129.2	-
			2’’’’’, 6’’’’’	128.7	7.90 (m)
			3’’’’’, 5’’’’’	127.5	7.13 (t, 7.4)
			4’’’’’	132.4	7.29 (t, 7.4)
			7’’’’’	166.3	-
4’-OMe	55.6	3.82 (s)	4’-OMe	54.6	3.88 (s)
7-OMe	56.6	3.87 (s)	7-OMe	55.4	3.83 (s)

^a^ Measured in methanol-*d*_4_, ^b^ 100 MHz, ^c^ 400 MHz, ^d^ 800 MHz, * overlapped signal. Assignments were done by HSQC, HMBC, and COSY experiments.

**Table 2:** NMR spectroscopic data for compounds **3** and **4**.

	3		4	
Pos.	*δ*_C_^a,b^	*δ*_H_^a,c^ (*J* in Hz)	*δ*_C_^a,b^	*δ*_H_^a,c^ (*J* in Hz)
1	56.6	4.27 * 4.09 *	56.6	4.33 * 4.11 *
3	174.9	-	174.7	-
4	35.9	2.77 (dd, 15.5, 5.5) 2.48 (dd, 15.5, 8.2)	35.8	2.78 (dd, 15.6, 5.7) 2.52 (dd, 15.6, 7.8)
5	49.2	3.17 (m)	49.9	3.24 (m)
6	76.5	3.95 (t, 5.0)	75.8	4.17 *
7	75.5	4.56 (d, 5.4)	78.3	5.87 (d, 5.8)
8	139.6	-	136.7	-
9	142.3	-	145.3	-
10	57.2	4.29 * 4.15 *	57.2	4.20 * 4.17 *
1’			122.5	-
2’, 6’			133.0	7.94 (d, 3.2)
3’, 5’			116.0	6.83 (d, 1.9)
4’			163.4	-
7’			168.1	-
3-OMe	52.1	3.75 (s)	52.2	3.58 (s)

^a^ Measured in methanol-*d*_4_, ^b^ 100 MHz, ^c^ 400 MHz, * overlapped signal. Assignments were done by HSQC, HMBC, COSY, and NOESY experiments.

**Table 3:** α-glucosidase activity of compounds (20 μM) isolated from *C. bignonioides*.

Compound	α-Glucosidase (units/L)
**Acarbose**	0.41 ± 0.25
**1**	0.82 ± 0.59
**2**	0.57 ± 0.64
**3**	0.43 ± 0.19
**4**	0.77 ± 0.11
**5**	1.31 ± 0.45
**6**	1.21 ± 0.15
**7**	1.41 ± 0.28
**8**	2.44 ± 0.32
**9**	0.41 ± 1.09
**10**	1.10 ± 1.03
**11**	1.08 ± 0.52
**12**	6.35 ± 1.70
**13**	0.67 ± 0.60
**14**	0.75 ± 0.35
**15**	0.67 ± 0.58

The absorbance was read at 405 nm using a microplate reader.

## Data Availability

The data presented in this study are available on request from the corresponding author.

## Supplementary Materials

The following are available online. Figures S1, S9 and S24: UV spectrum of compound **1**, **2** and **4**, Figures S2, S10, S18 and S25: IR spectrum of compound **1**, **2**, **3** and **4**, Figures S3 and S11: CD spectrum of compound **1** and **2**, Figures S4, S12, S19, S26: ^1^H-NMR spectrum of compound **1**, **2**, **3** and **4**, Figures S5, S13, S20, S27: ^13^C-NMR spectrum of compound **1**, **2**, **3** and **4**, Figures S6, S7, S14, S15, S21, S22, S28 and S29: HSQC and HMBC spectrum of compound **1**, **2**, **3** and **4**, Figures S8, S16, S23 and S30: HR-ESI-MS spectrum of compound **1**, **2**, **3** and **4**, Figure S17: MS/MS spectrum of compound **2**.

## Sample Availability

Compounds used in this study are not available from the authors.
