# Supplementary material for: The Chemical Constituents from Fruits of Catalpa bignonioides Walt. and Their α-Glucosidase Inhibitory Activity and Insulin Secretion Effect
Source: Molecules. 2021 Jan 12;26(2):362. doi: 10.3390/molecules26020362 (PMC7826643; doi:10.3390/molecules26020362)
Supplement: Supplementary file 1 [file molecules-26-00362-s001.pdf]

Supporting Information for

The chemical constituents from fruits of *Catalpa bignonioides* Walt. and their α-glucosidase inhibitory activity and insulin secretion effect

Youngse Oh[1], Dahae Lee [2], SeonJu Park[1,3], Seung Hyun Kim[1,*] , Ki Sung Kang[2,*]

[1] College of Pharmacy, Yonsei Institute of Pharmaceutical Sciences, Yonsei University, Incheon 21983, South Korea
[2] College of Korean Medicine, Gachon University, Seongnam 13120, South Korea
[3] Chuncheon Center, Korea Basic Science Institute (KBSI), Chuncheon 24341, South Korea

Corresponding authors:
*(K.S.K.) Tel: +82-31-750-5402. E-mail: kkang@gachon.ac.kr
*(S.H.K.) Tel: +82-32-749-4514. E-mail: kimsh11@yonsei.ac.kr

List of Contents

no.	Content	Page
1	**Figure S1.** UV spectrum of compound **1**	S4
2	**Figure S2.** IR spectrum of compound **1**	S5
3	**Figure S3.** CD spectrum of compound **1**	S6
4	**Figure S4.** ^{1}H-NMR spectrum of compound **1**	S7
5	**Figure S5.** ^{13}C-NMR spectrum of compound **1**	S8
6	**Figure S6.** HSQC spectrum of compound **1**	S9
7	**Figure S7.** HMBC spectrum of compound **1**	S10
8	**Figure S8.** HR-ESI-MS of compound **1**	S11
9	**Figure S9.** UV spectrum of compound **2**	S12
10	**Figure S10.** IR spectrum of compound **2**	S13
11	**Figure S11.** CD spectrum of compound **2**	S14
12	**Figure S12.** ^{1}H-NMR spectrum of compound **2**	S15
13	**Figure S13.** ^{13}C-NMR spectrum of compound **2**	S16
14	**Figure S14.** HSQC spectrum of compound **2**	S17
15	**Figure S15.** HMBC spectrum of compound **2**	S18
16	**Figure S16.** HR-ESI-MS of compound **2**	S19
17	**Figure S17.** MS/MS spectrum of compound **2**	S20
18	**Figure S18.** IR spectrum of compound **3**	S21
19	**Figure S19.** ^{1}H-NMR spectrum of compound **3**	S22
20	**Figure S20.** ^{13}C-NMR spectrum of compound **3**	S23

21	**Figure S21.** HSQC spectrum of compound **3**	S24
22	**Figure S22.** HMBC spectrum of compound **3**	S25
23	**Figure S23.** HR-ESI-MS spectrum of compound **3**	S26
24	**Figure S24.** UV spectrum of compound **4**	S27
25	**Figure S25.** IR spectrum of compound **4**	S28
26	**Figure S26.** ^{1}H-NMR spectrum of compound **4**	S29
27	**Figure S27.** ^{13}C-NMR spectrum of compound **4**	S30
28	**Figure S28.** HSQC spectrum of compound **4**	S31
29	**Figure S29.** HMBC spectrum of compound **4**	S32
30	**Figure S30.** HR-ESI-MS spectrum of compound **4**	S33

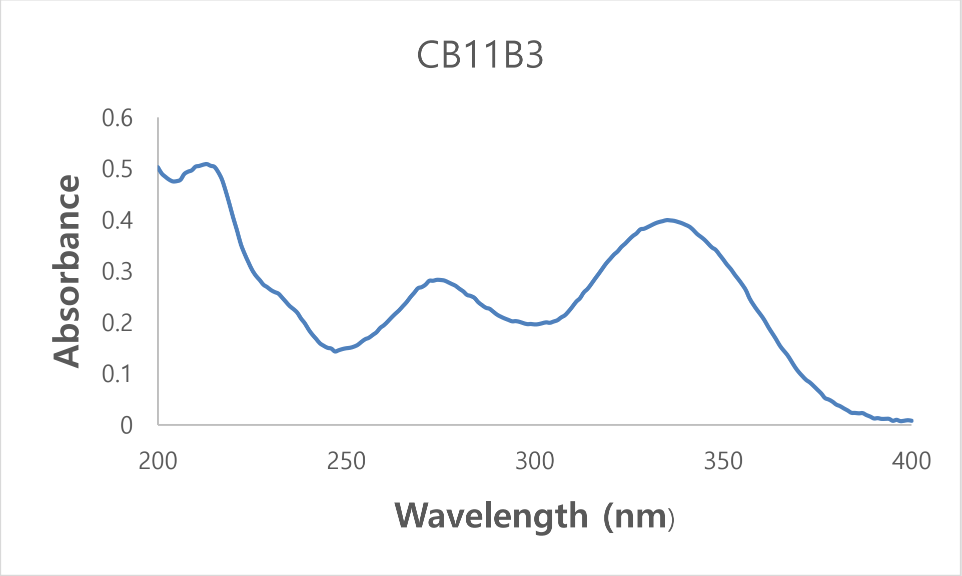

Figure S1. UV spectrum of compound **1**

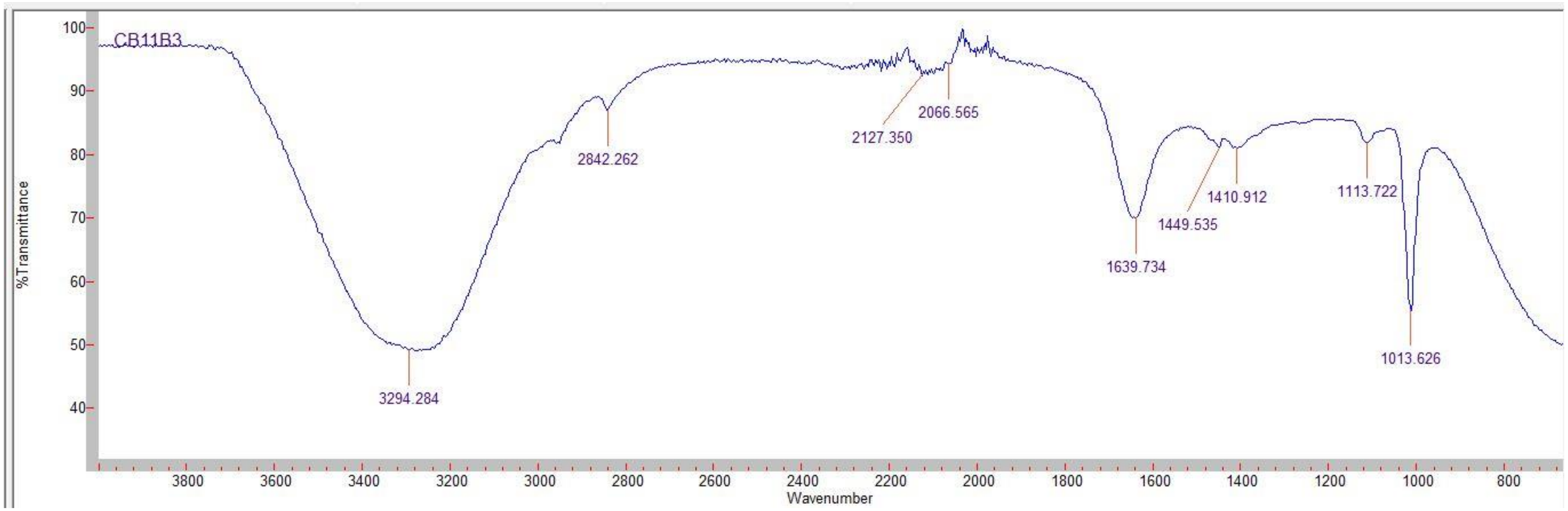

Figure S2. IR spectrum of compound **1**

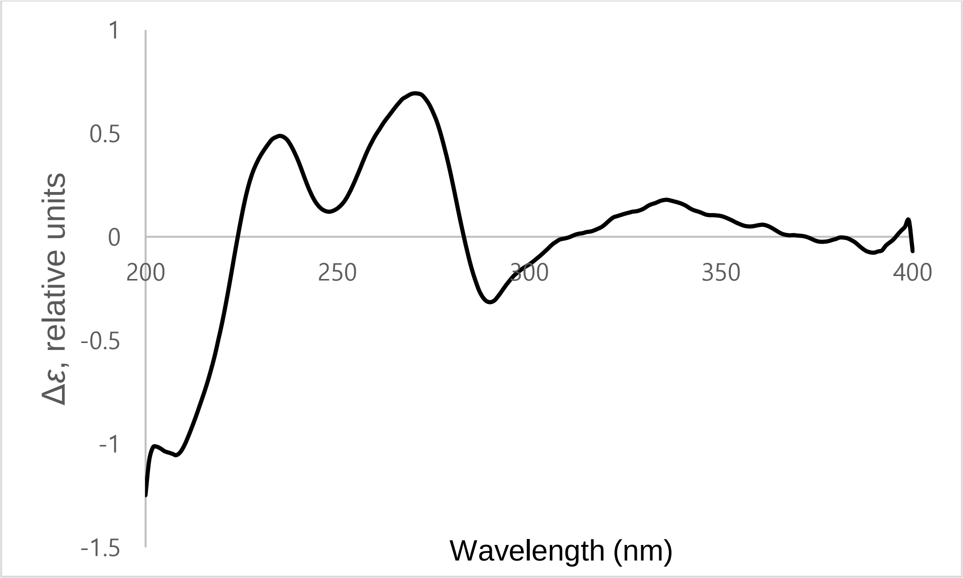

Figure S3. CD spectrum of compound **1**

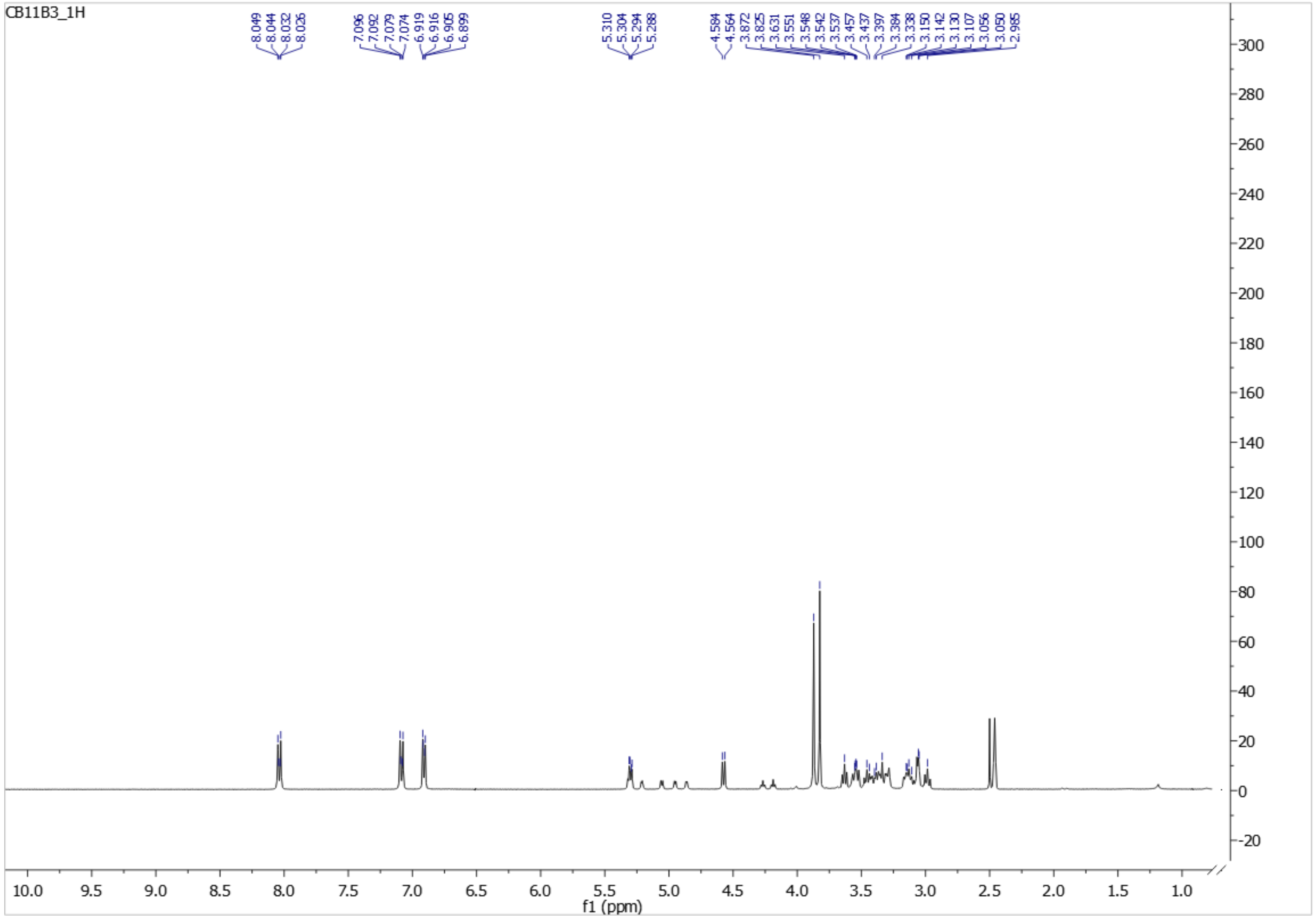

Figure S4. ^{1}H-NMR spectrum of compound **1** (400 MHz, Dimethyl sulfoxide-d_6)

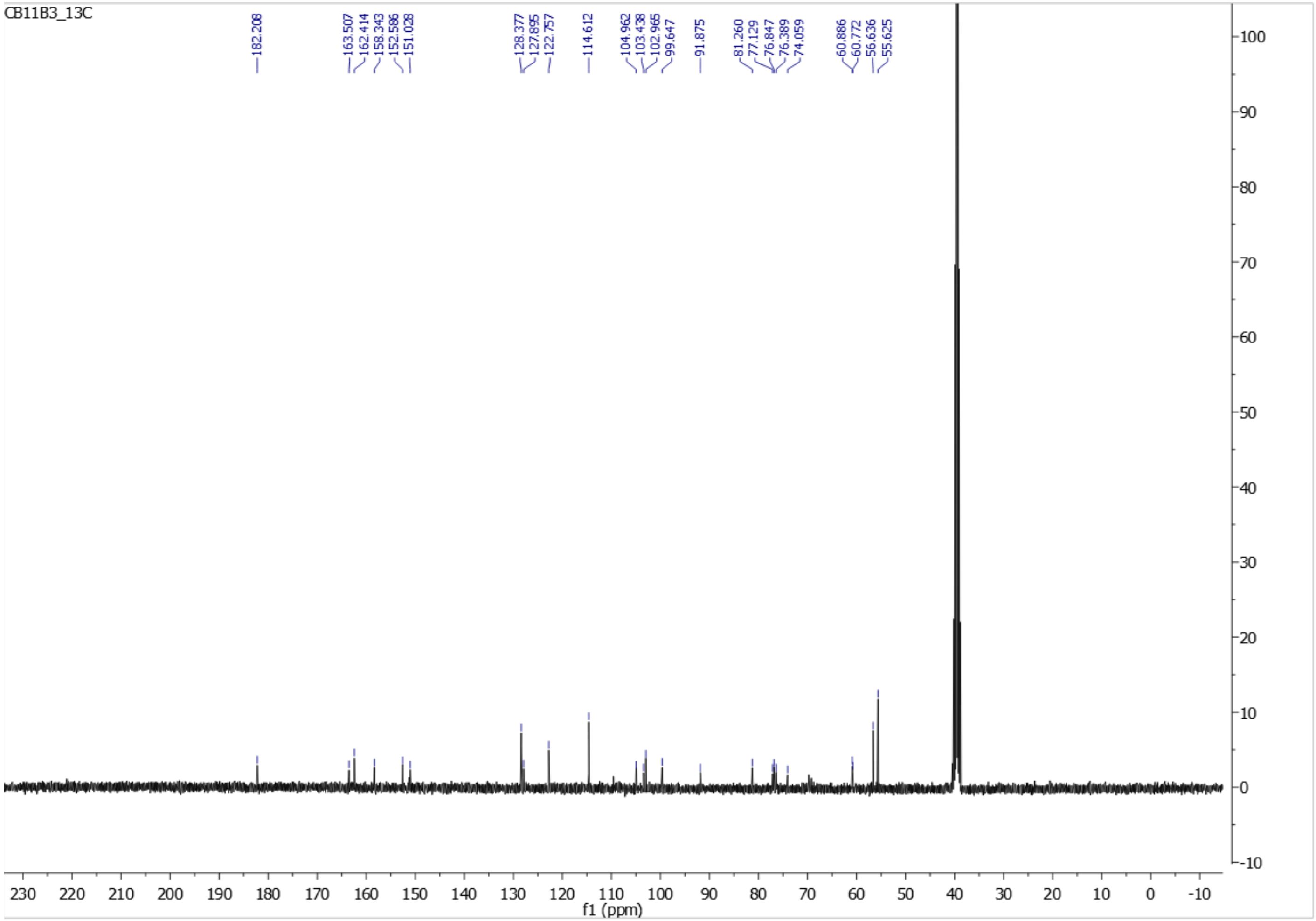

Figure S5. ^{13}C-NMR spectrum of compound **1** (100 MHz, Dimethyl sulfoxide-d_6)

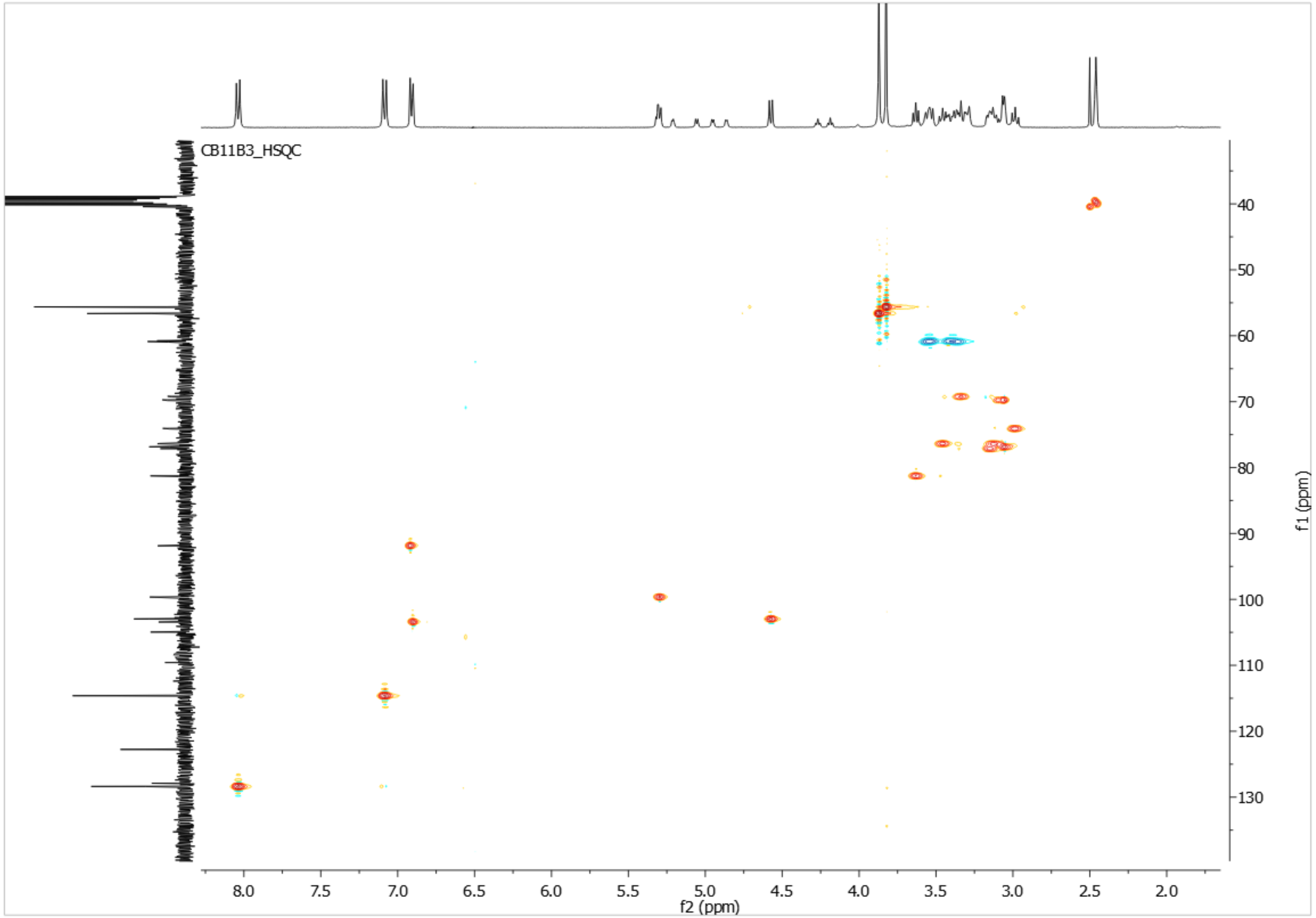

Figure S6. HSQC spectrum of compound **1** (Dimethyl sulfoxide-d_6)

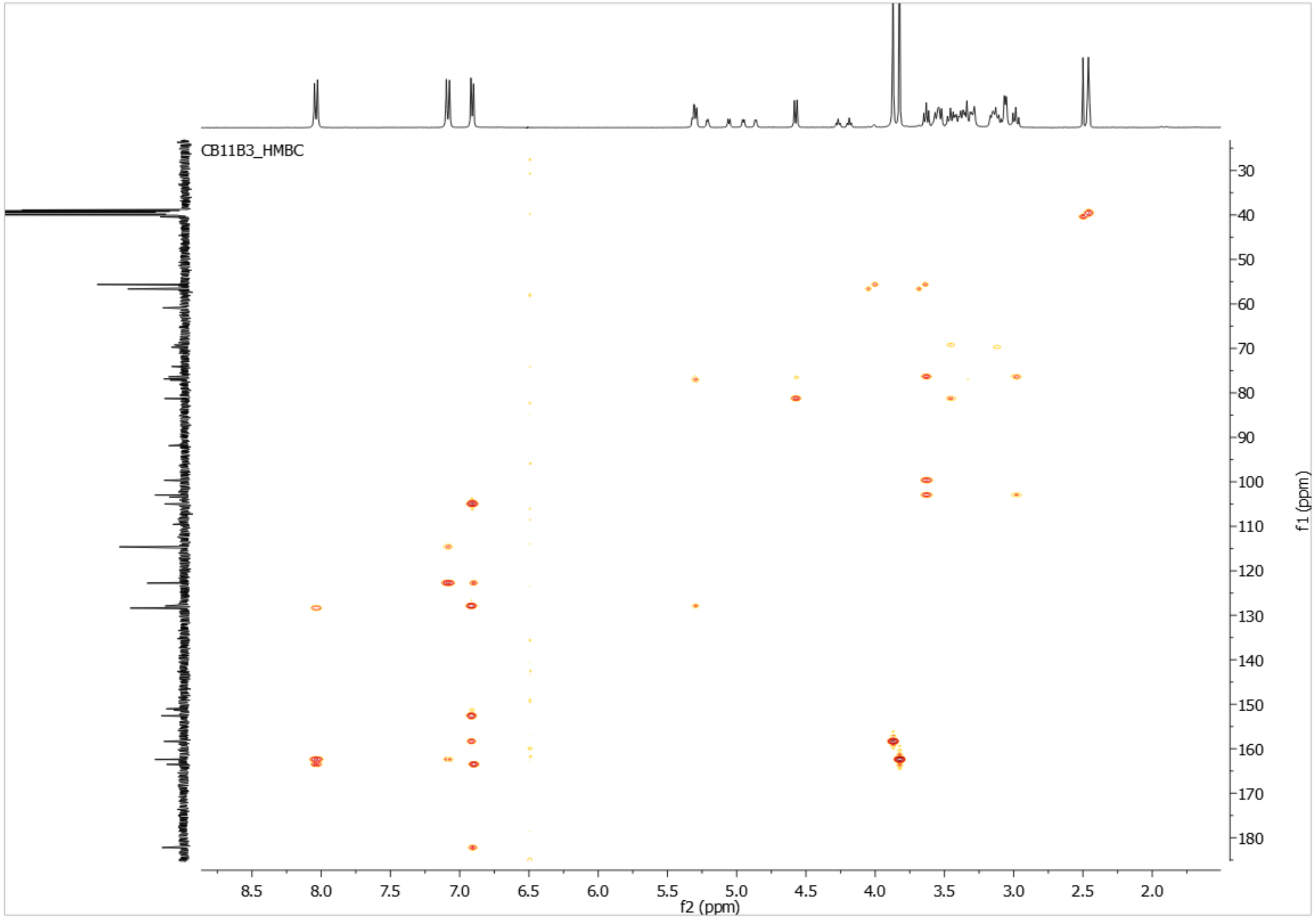

Figure S7. HMBC spectrum of compound **1** (Dimethyl sulfoxide-d_6)

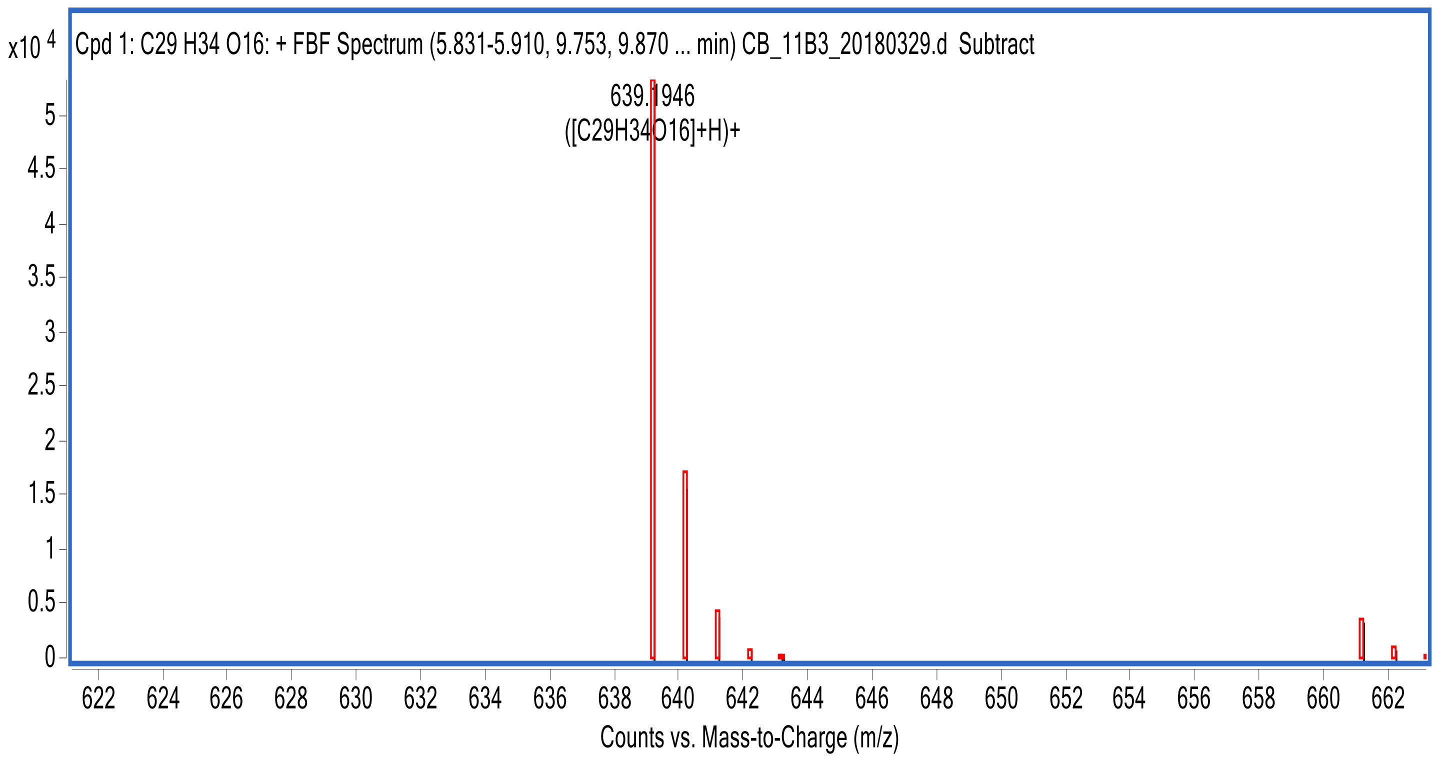

Figure S8. HR-ESI-MS of compound **1**

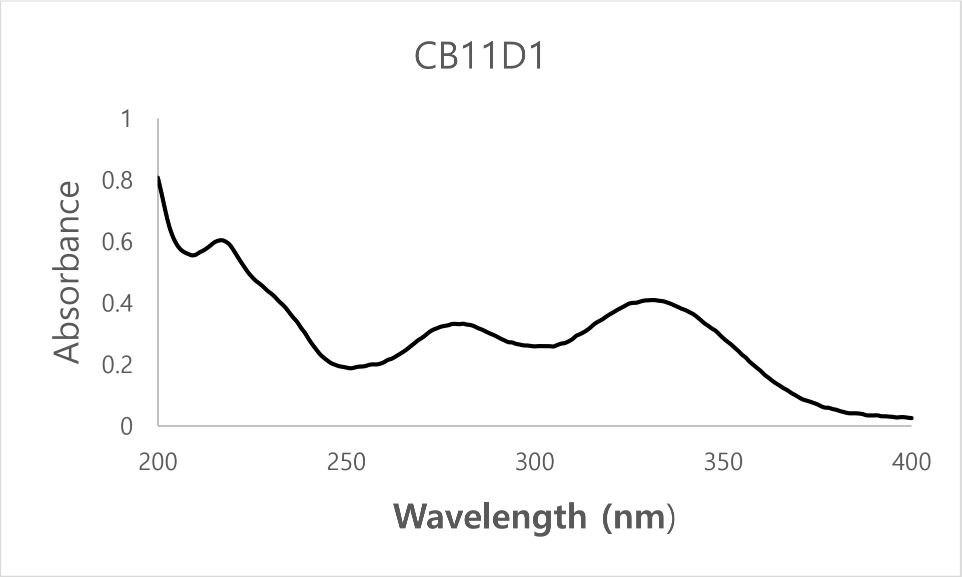

Figure S9. UV spectrum of compound **2**

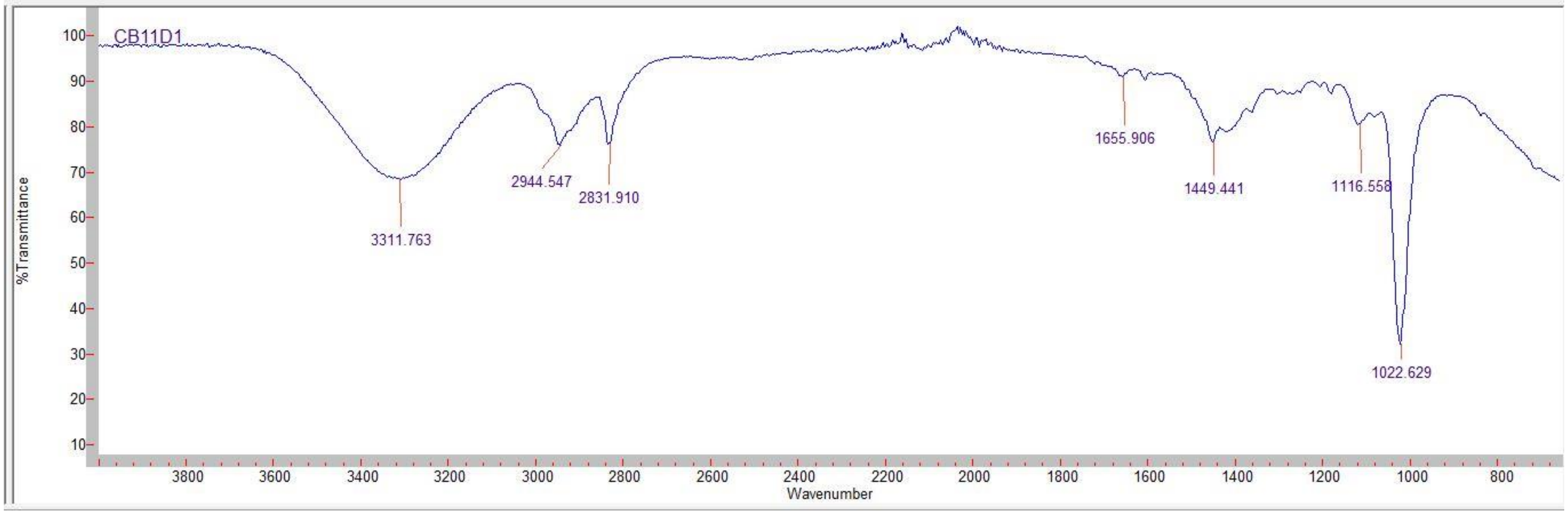

Figure S10. IR spectrum of compound **2**

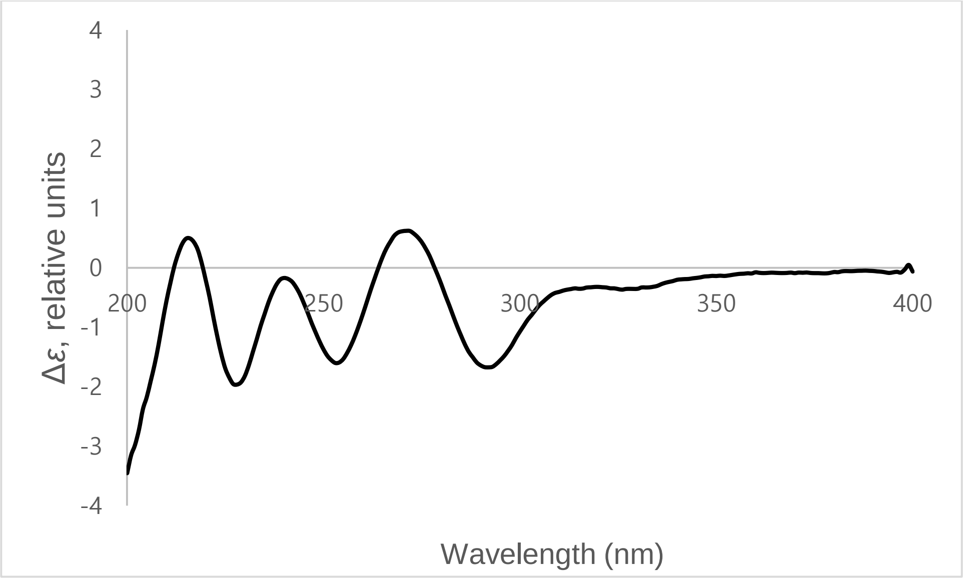

Figure S11. CD spectrum of compound **2**

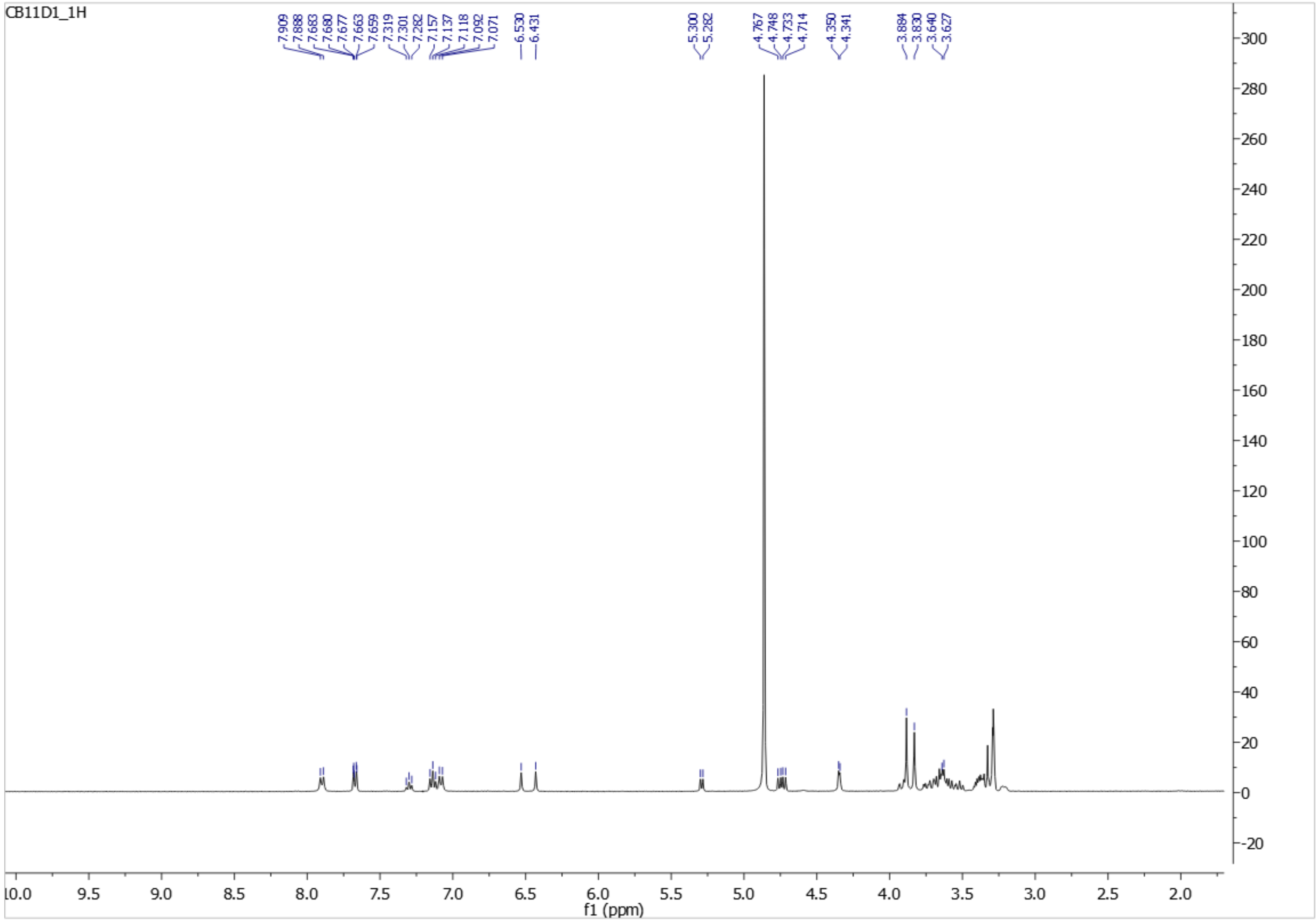

Figure S12. ^{1}H-NMR spectrum of compound **2** (400 MHz, methanol-d_4)

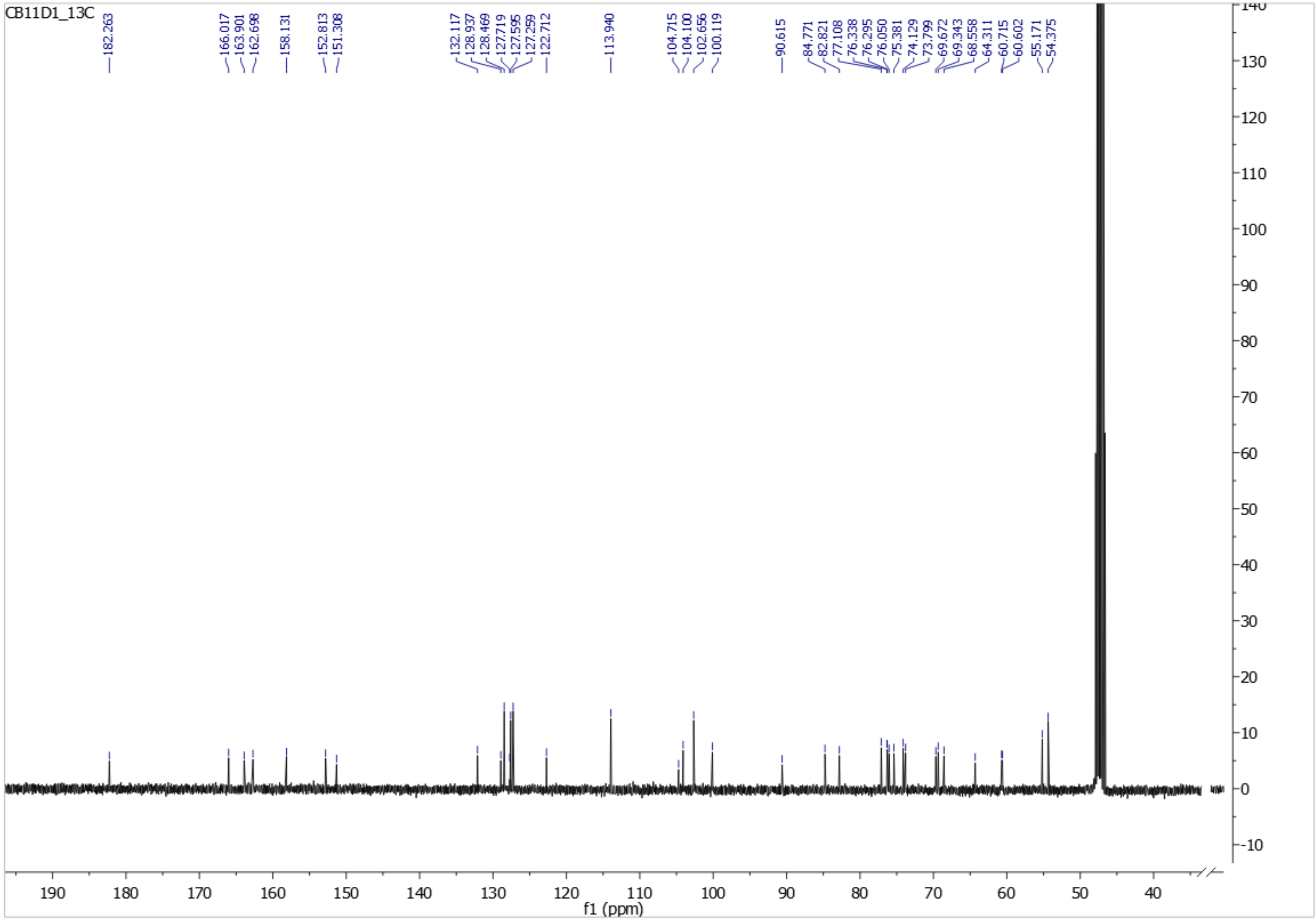

Figure S13. ^{13}C-NMR spectrum of compound **2** (100 MHz, methanol-d_4)

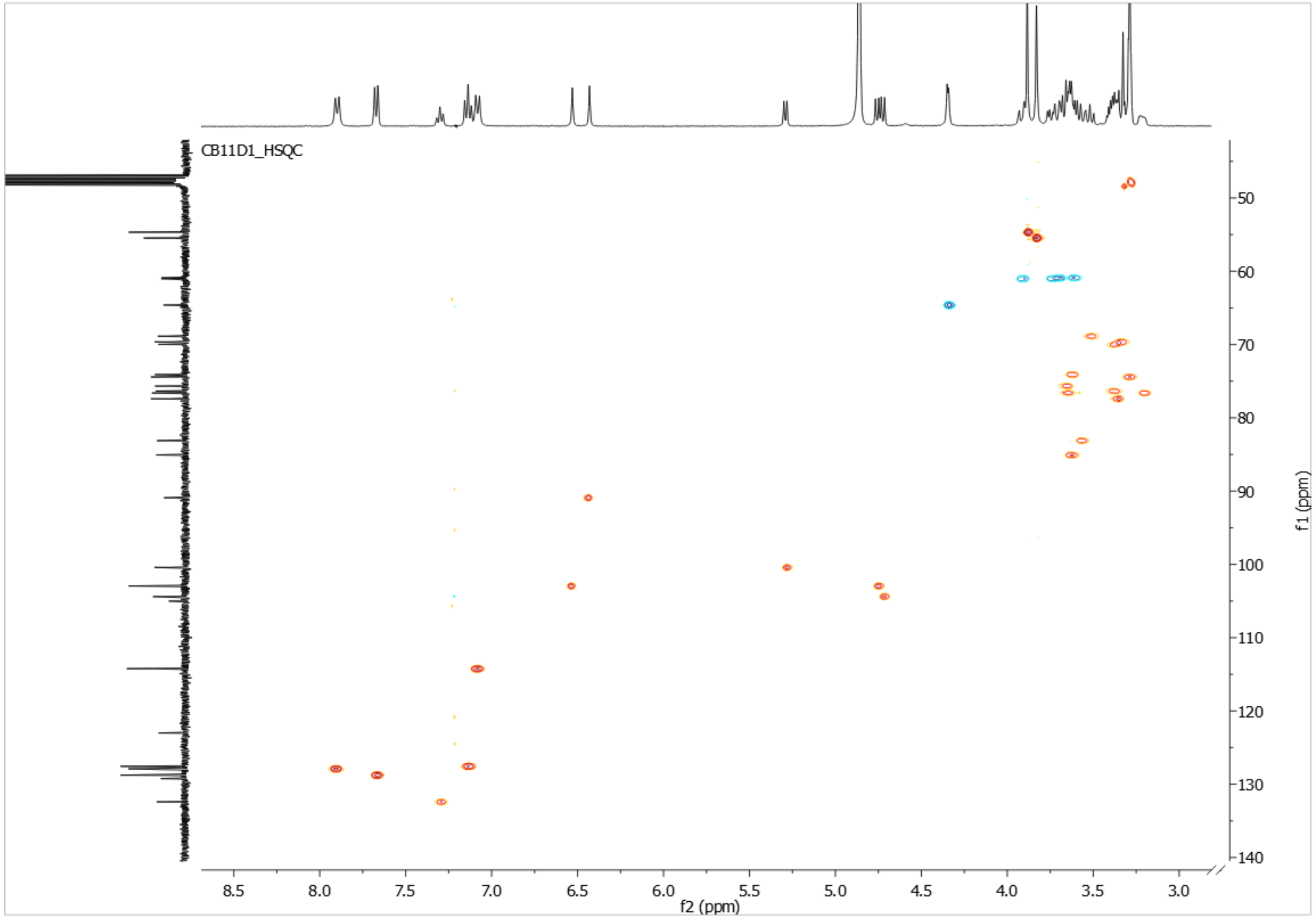

Figure S14. HSQC spectrum of compound **2** (methanol-d_4)

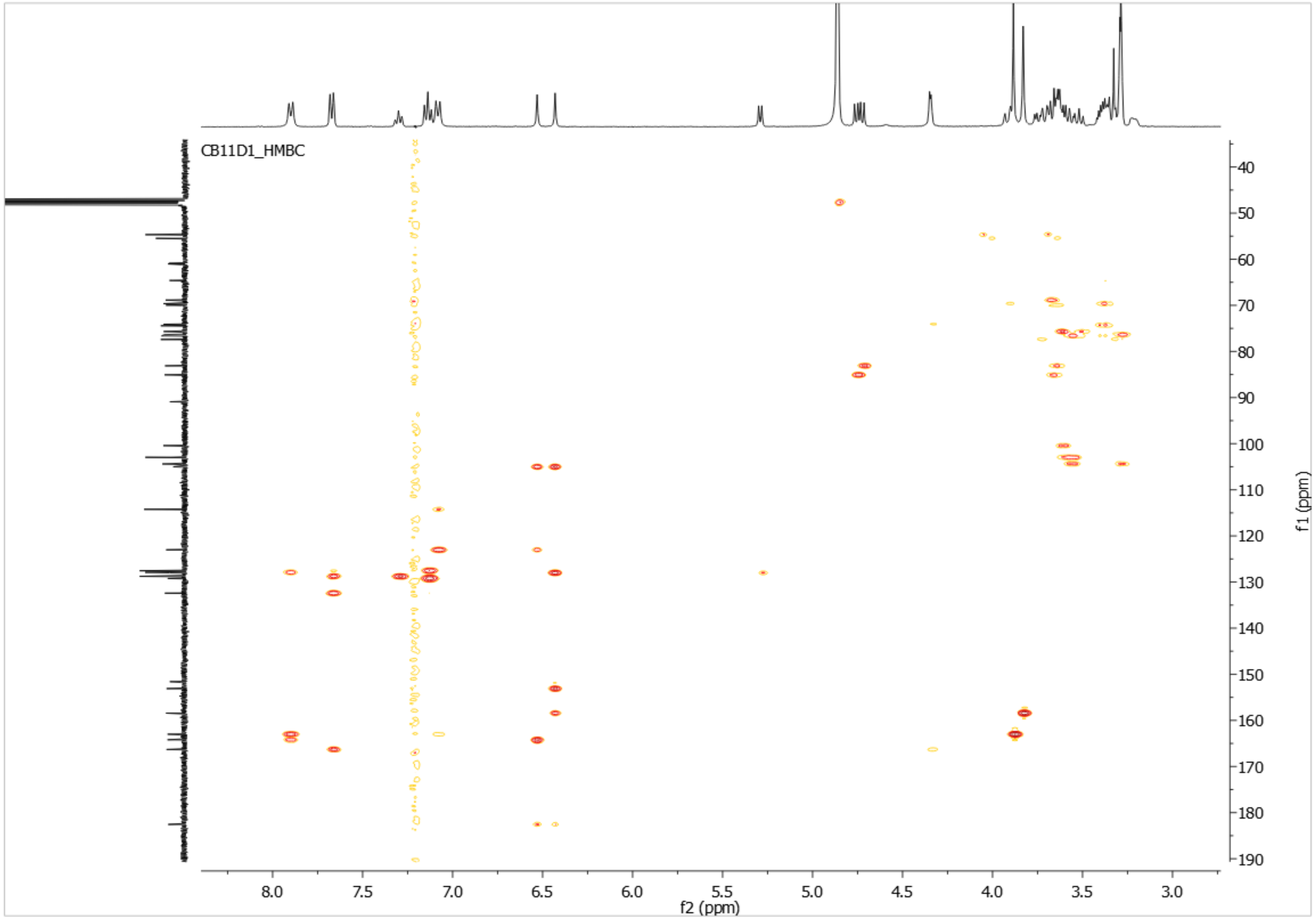

Figure S15. HMBC spectrum of compound **2** (methanol-d_4)

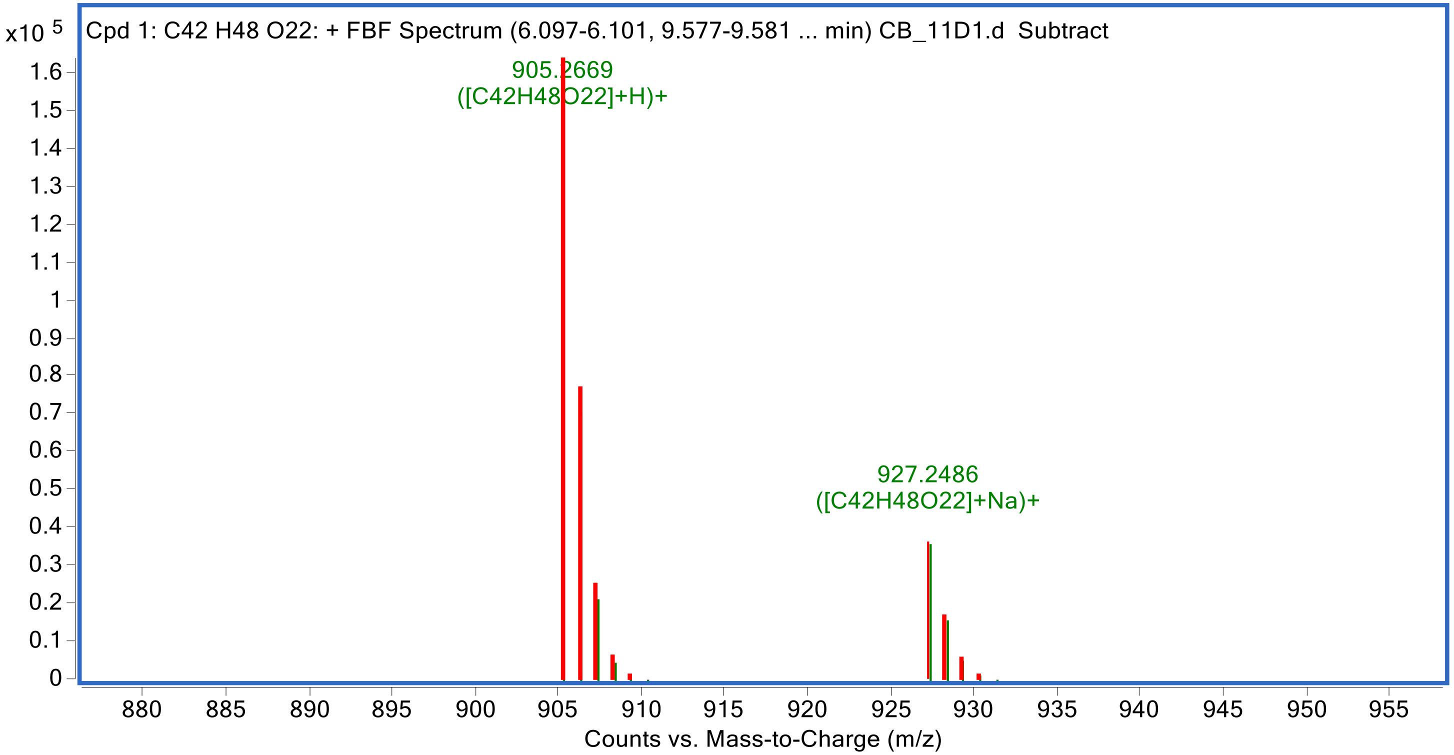

Figure S16. HR-ESI-MS of compound **2**

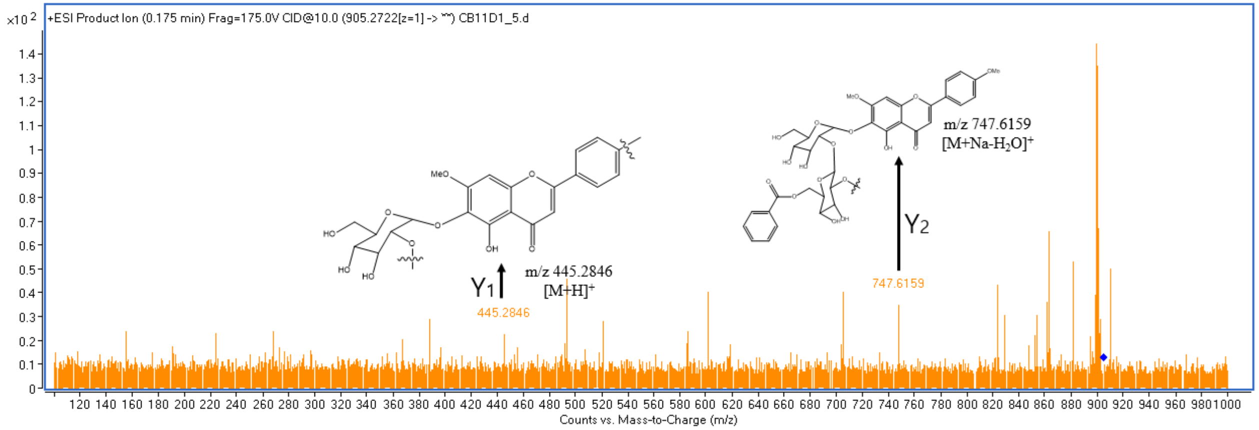

The ms/ms spectrum of compound 2 (*m/z* 905.2772)

Y1 : $[M+H]^+$ = 445.2846

Y2 : $[M+Na-H_2O]^+$ = 747.6159

Figure S17. MS/MS spectrum of compound **2**

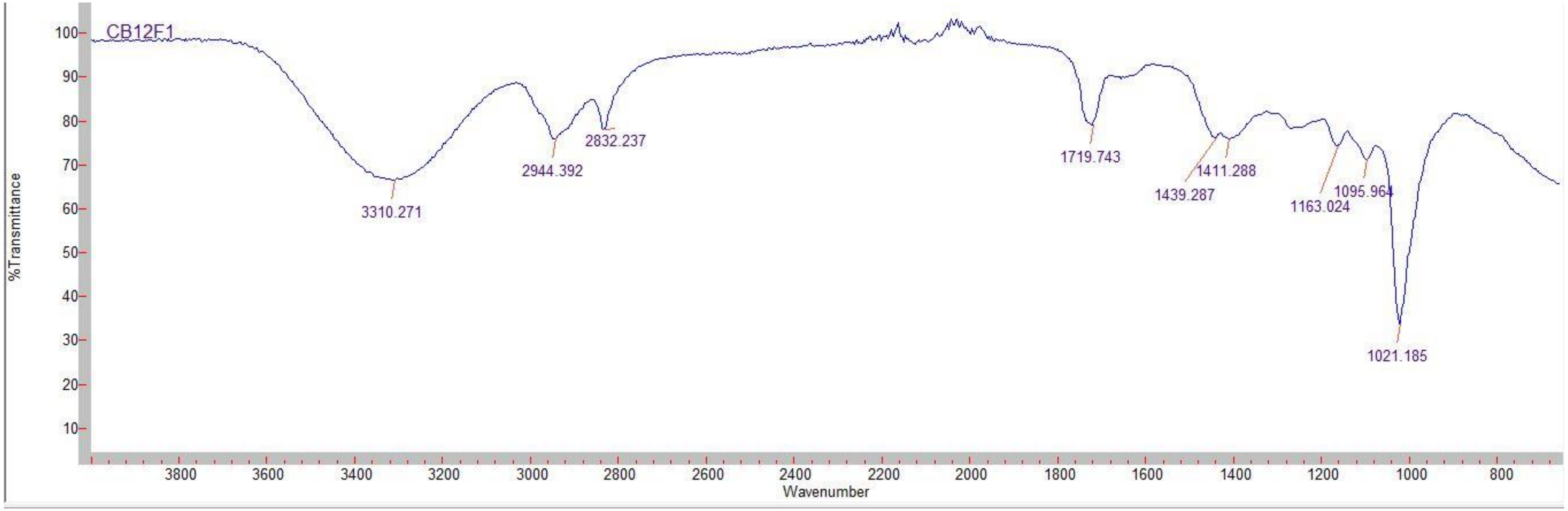

Figure S18. IR spectrum of compound **3**

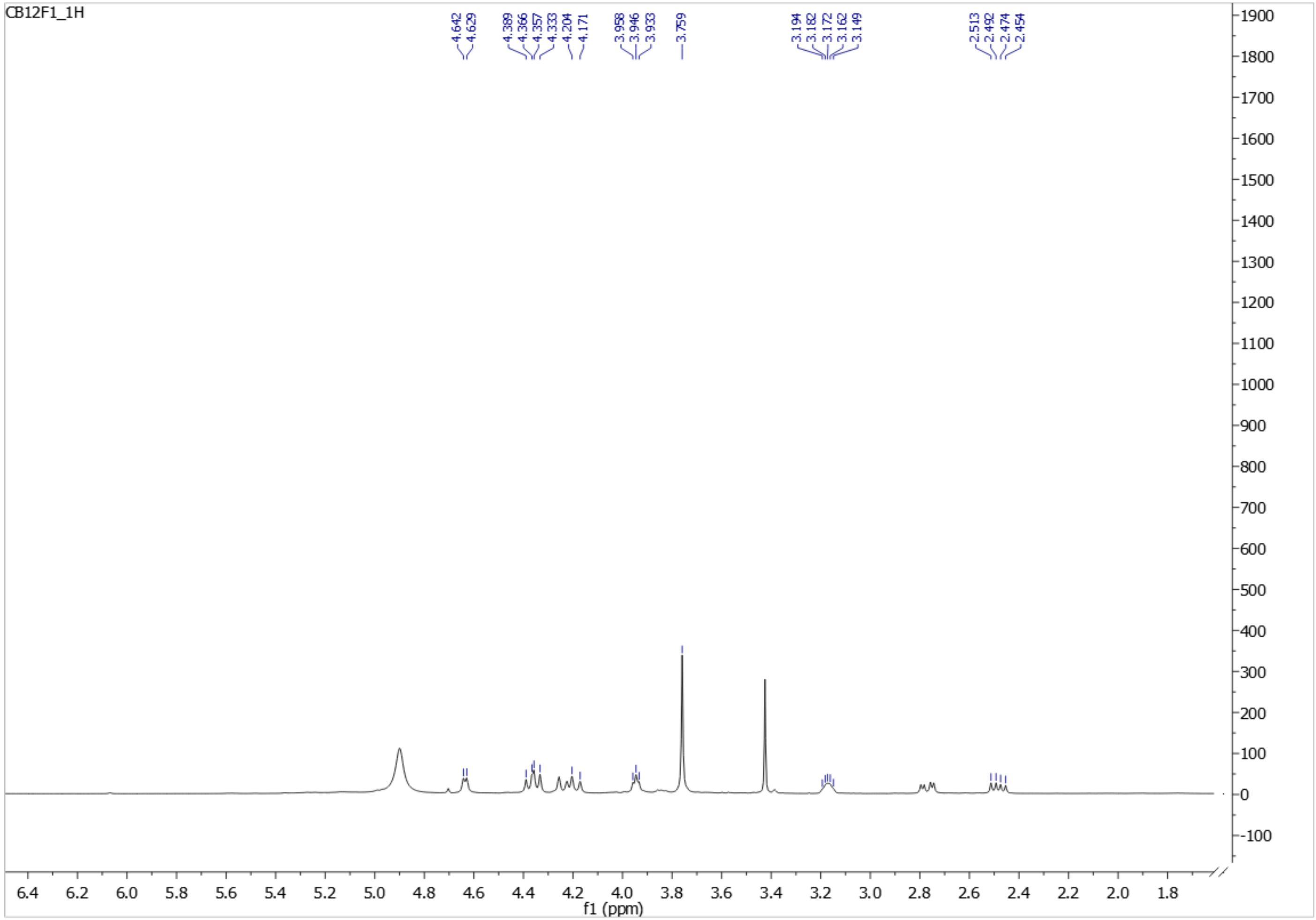

Figure S19. ^{1}H-NMR spectrum of compound **3** (400 MHz, methanol-d_4)

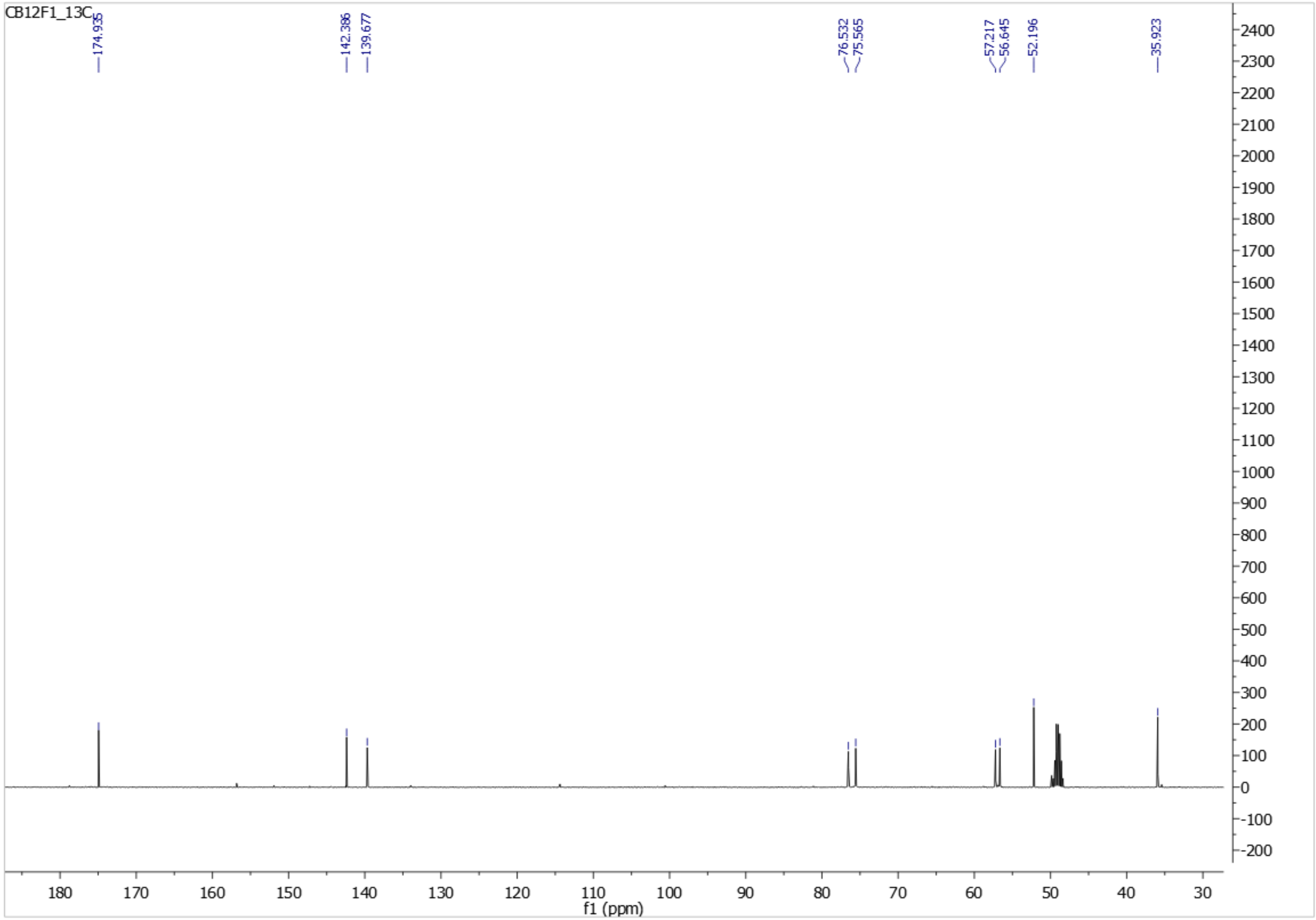

Figure S20. ^{13}C-NMR spectrum of compound **3** (100 MHz, methanol-d_4)

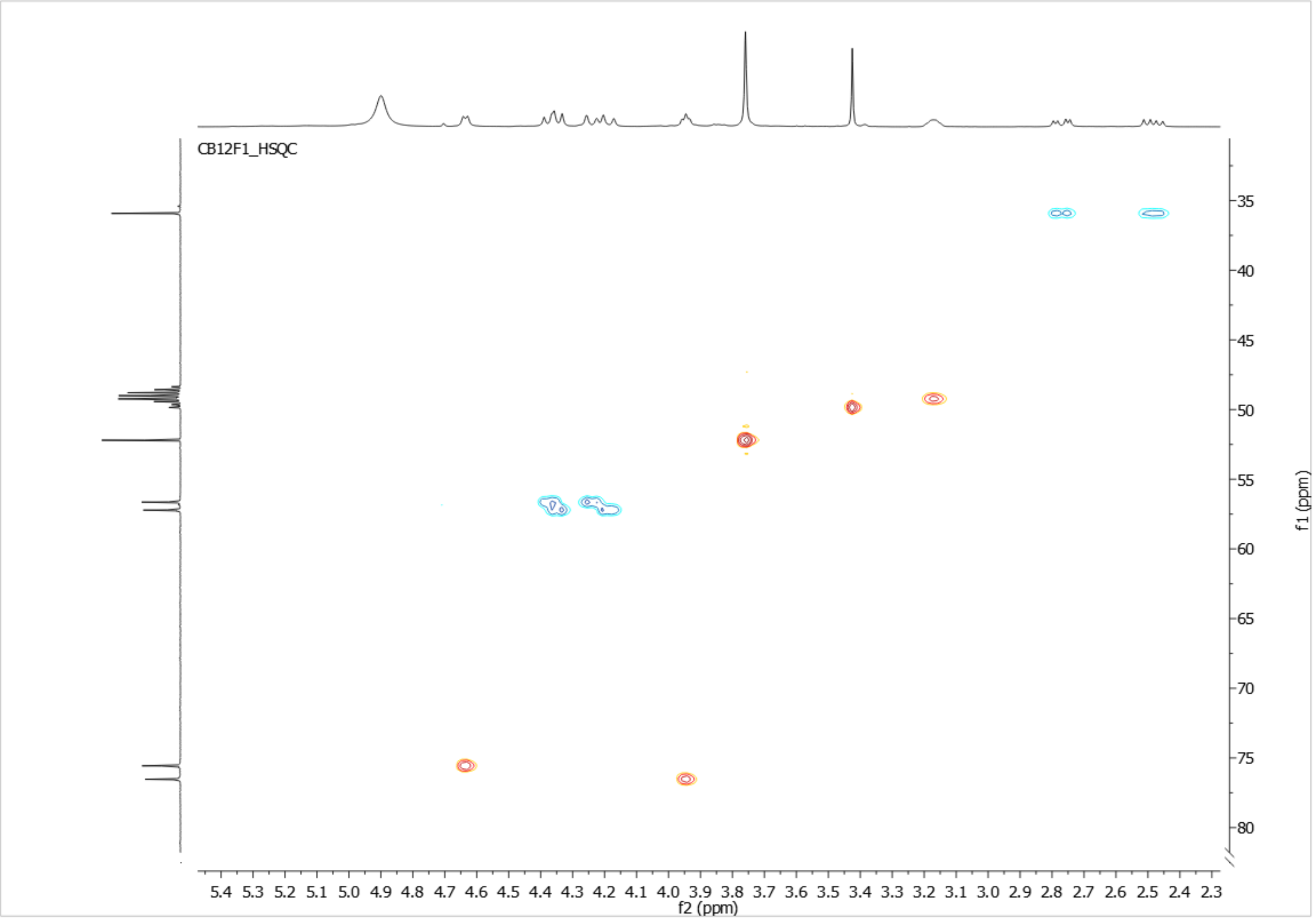

Figure S21. HSQC spectrum of compound **3** (methanol-d_4)

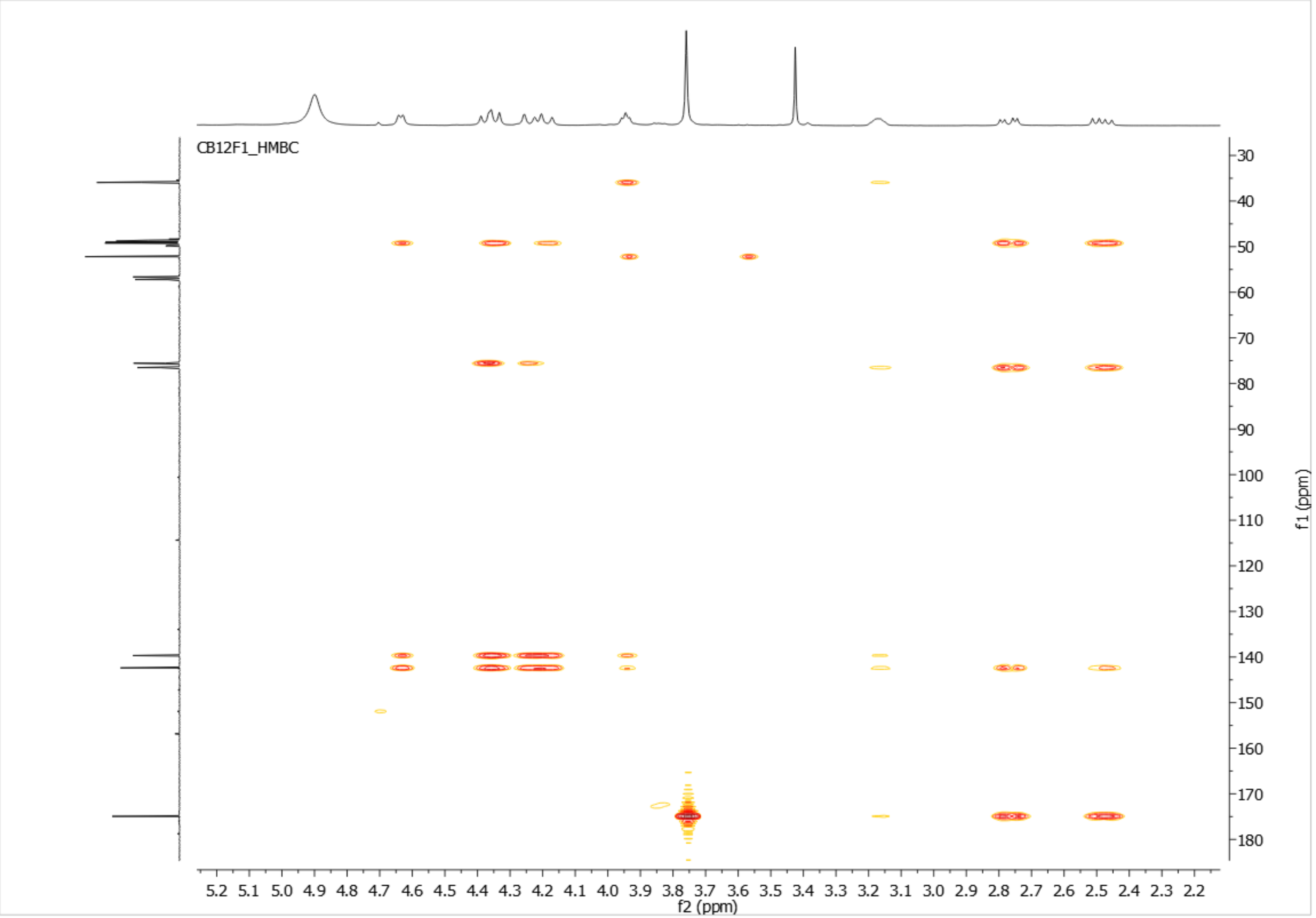

Figure S22. HMBC spectrum of compound **3** (methanol-d_4)

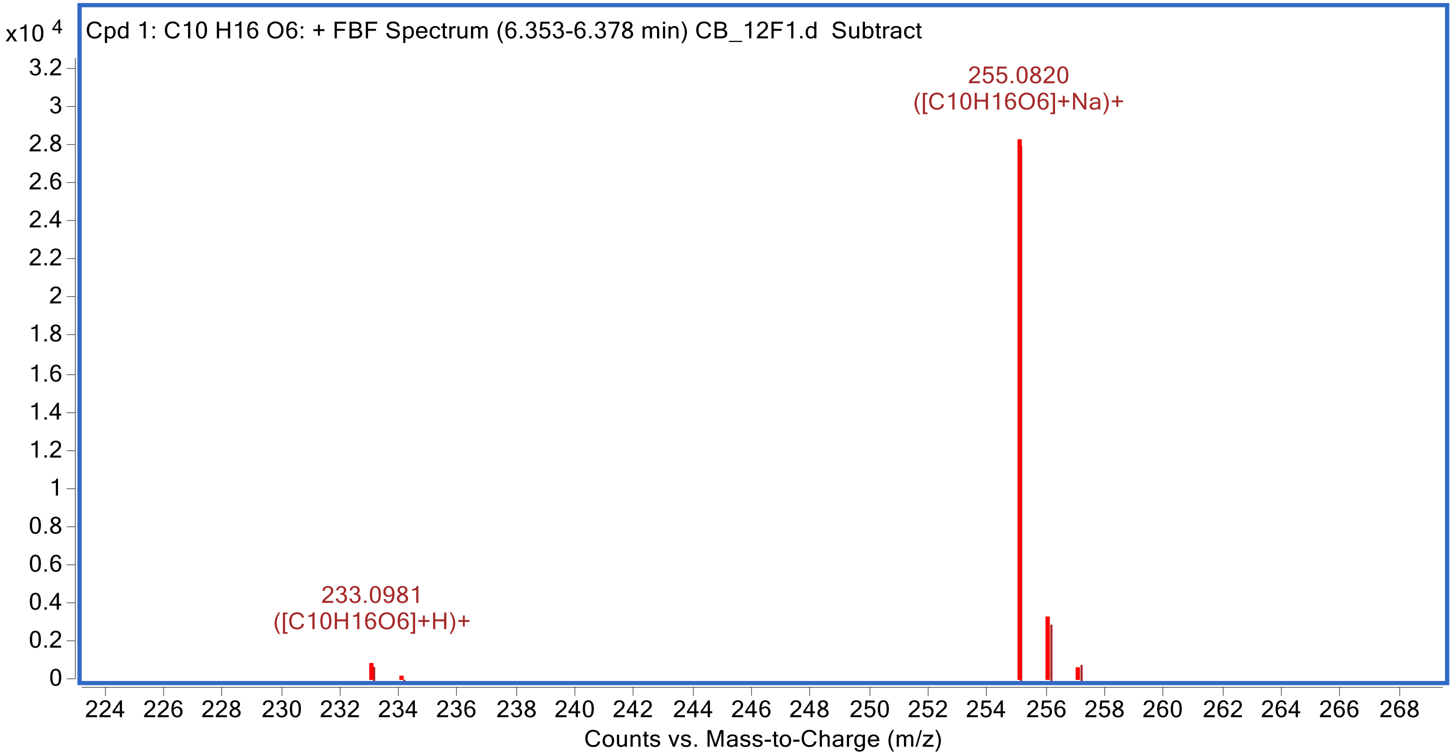

Figure S23. HR-ESI-MS of compound **3**

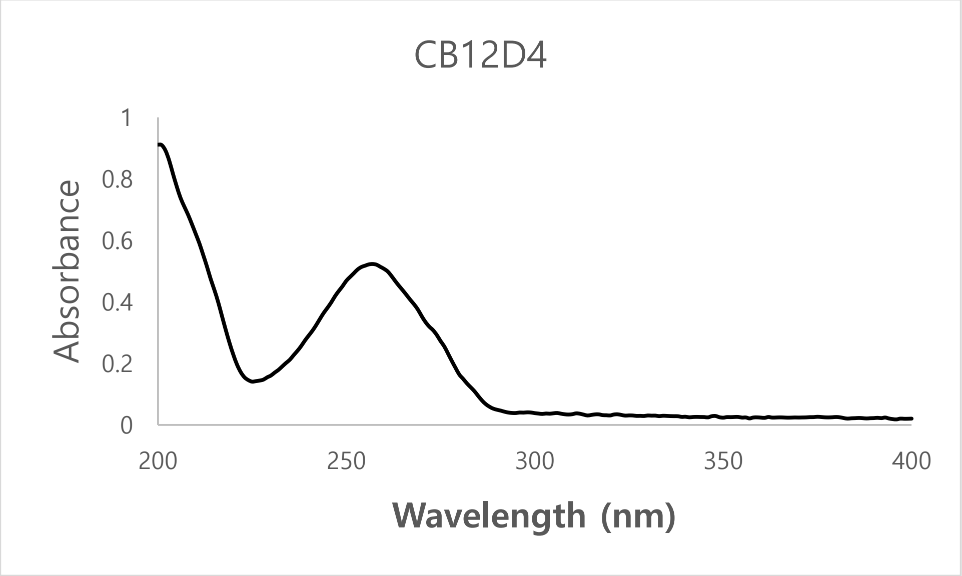

Figure S24. UV spectrum of compound **4**

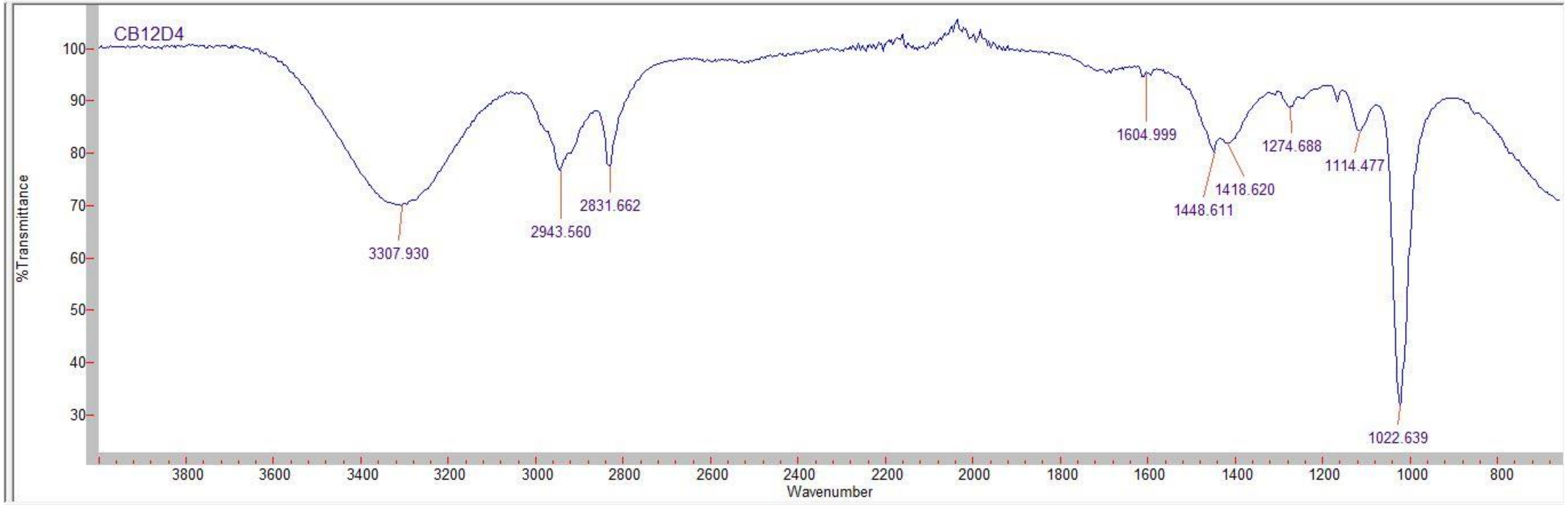

Figure S25. IR spectrum of compound **4**

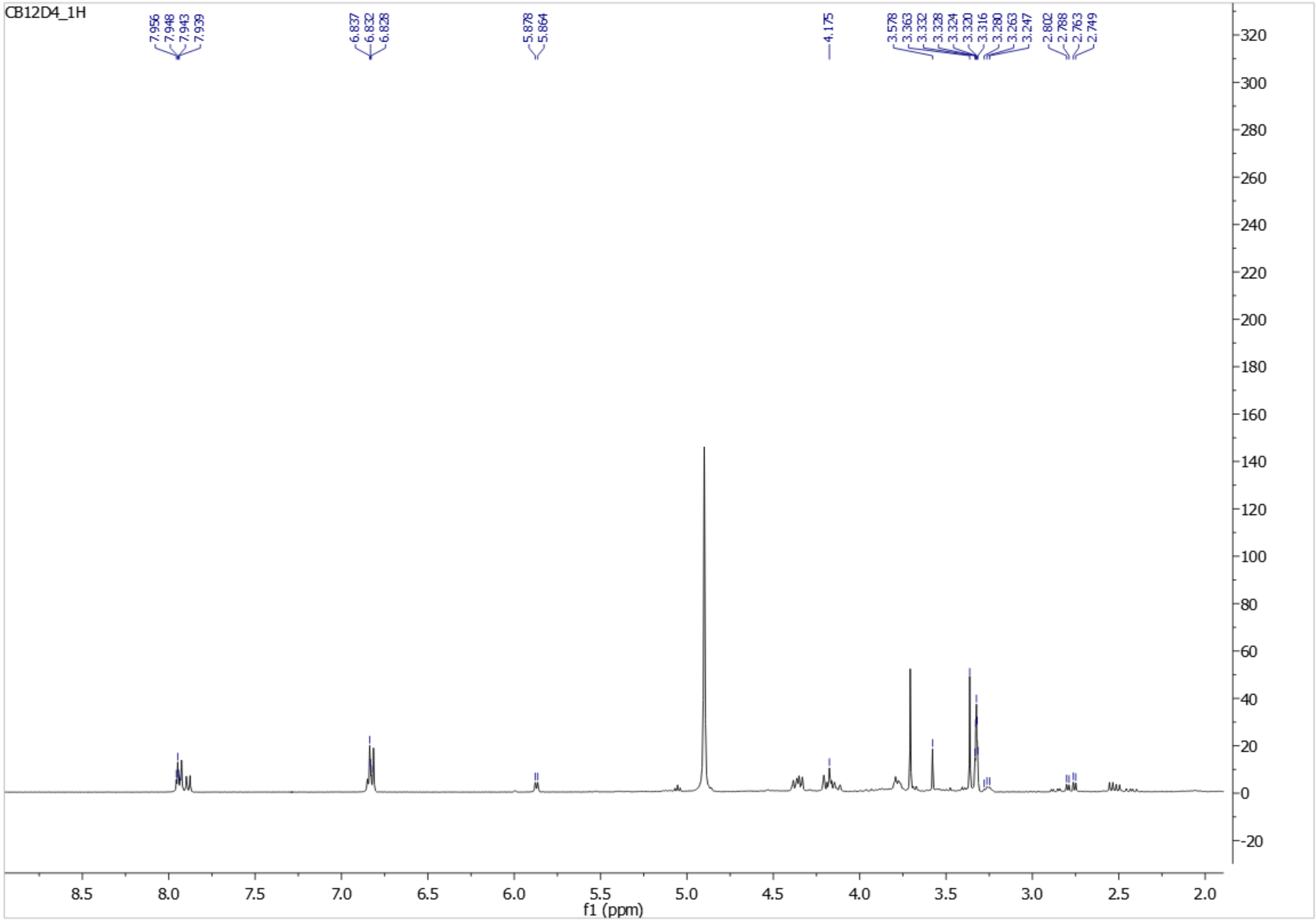

Figure S26. ^{1}H-NMR spectrum of compound **4** (400 MHz, methanol-d_4)

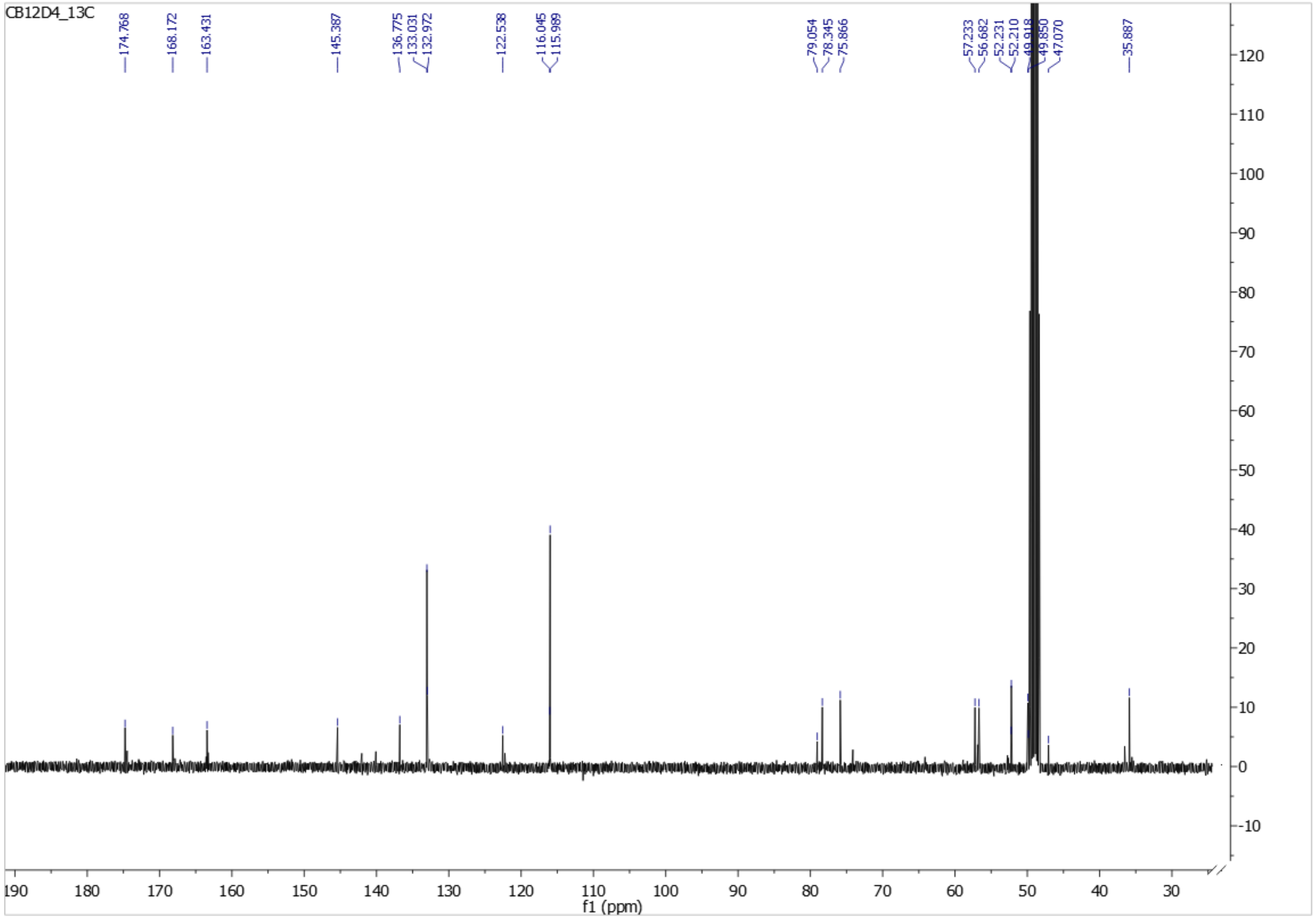

Figure S27. ^{13}C-NMR spectrum of compound **4** (100 MHz, methanol-d_4)

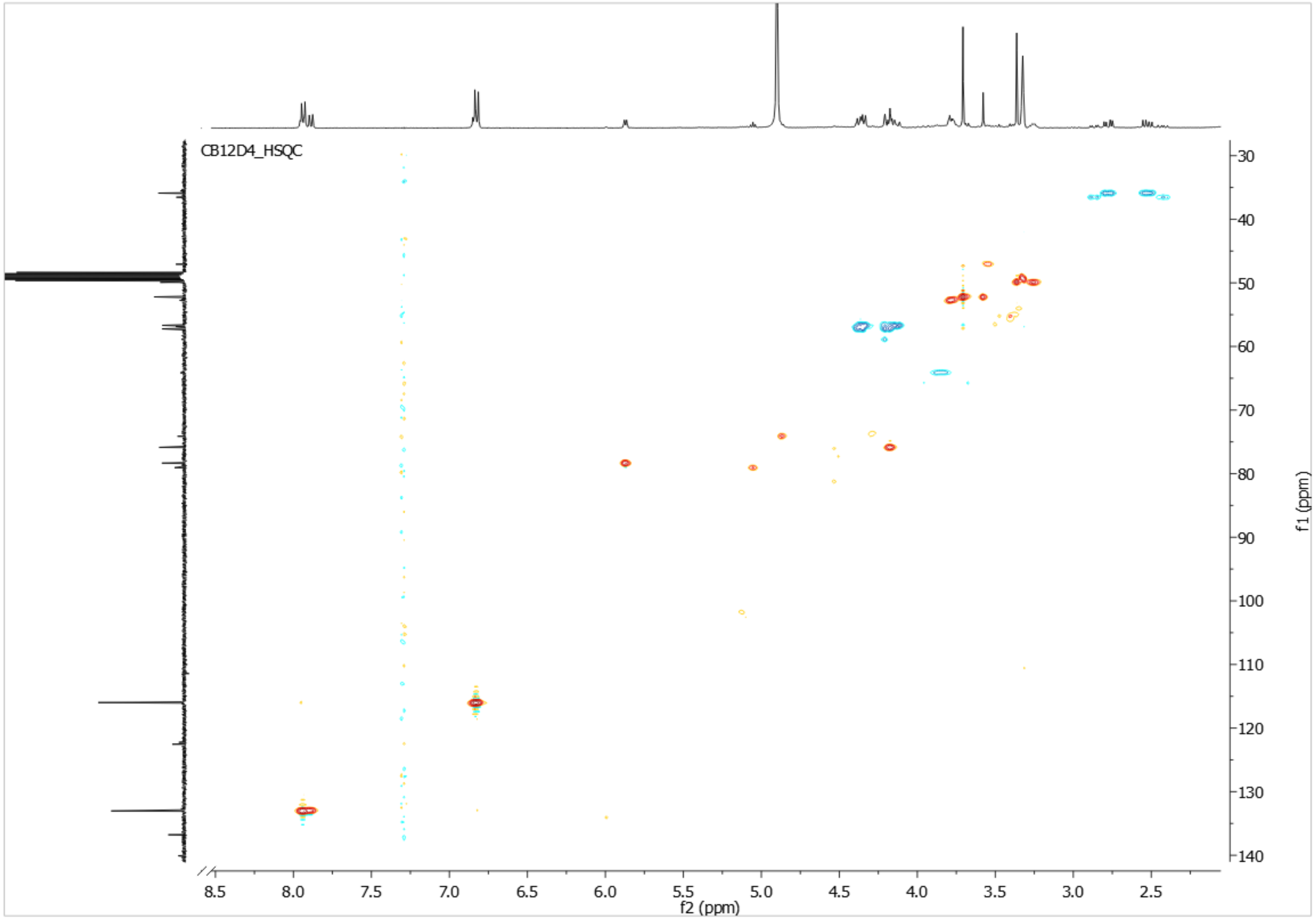

Figure S28. HSQC spectrum of compound **4** (methanol-d_4)

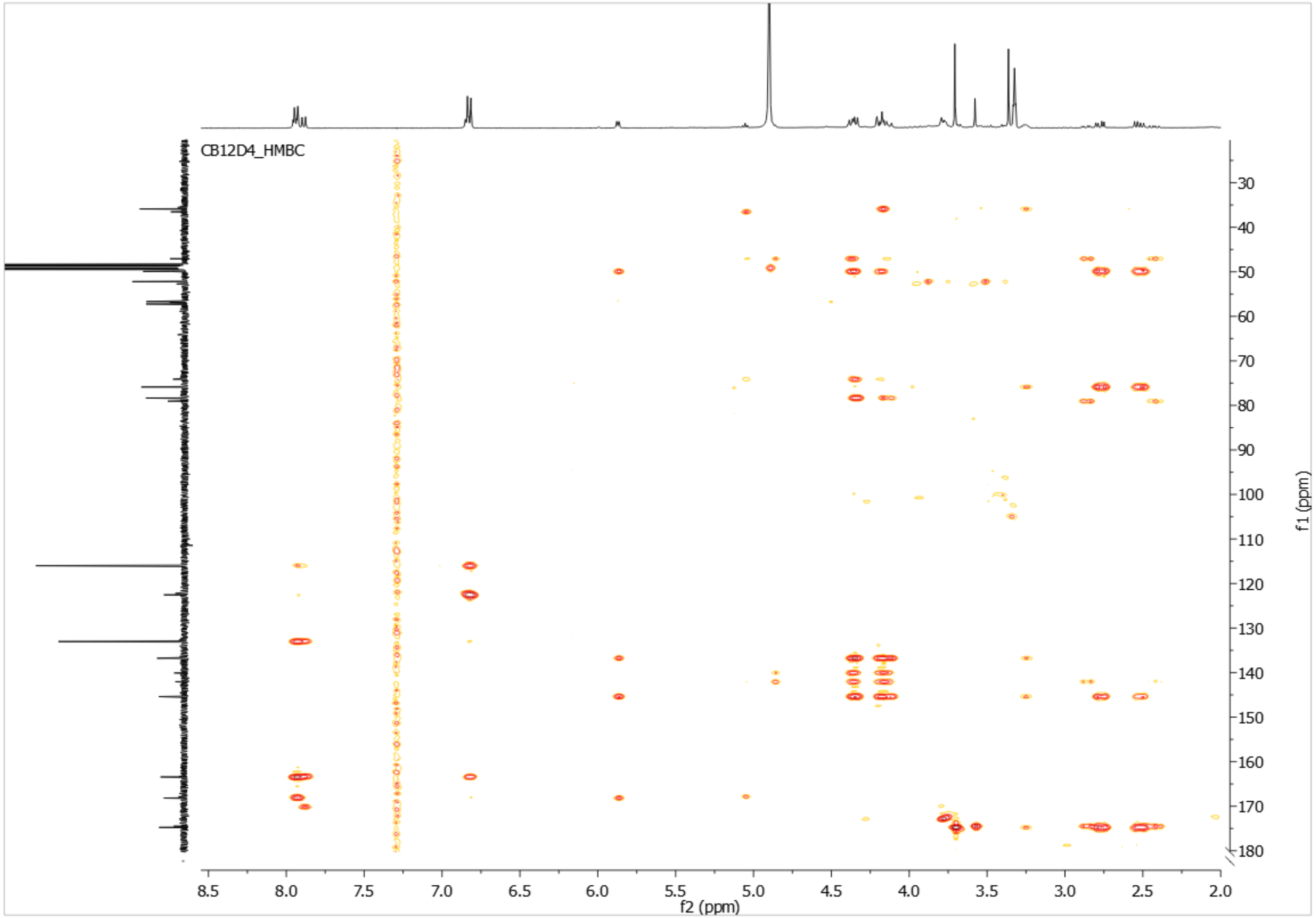

Figure S29. HMBC spectrum of compound **4** (methanol-d_4)

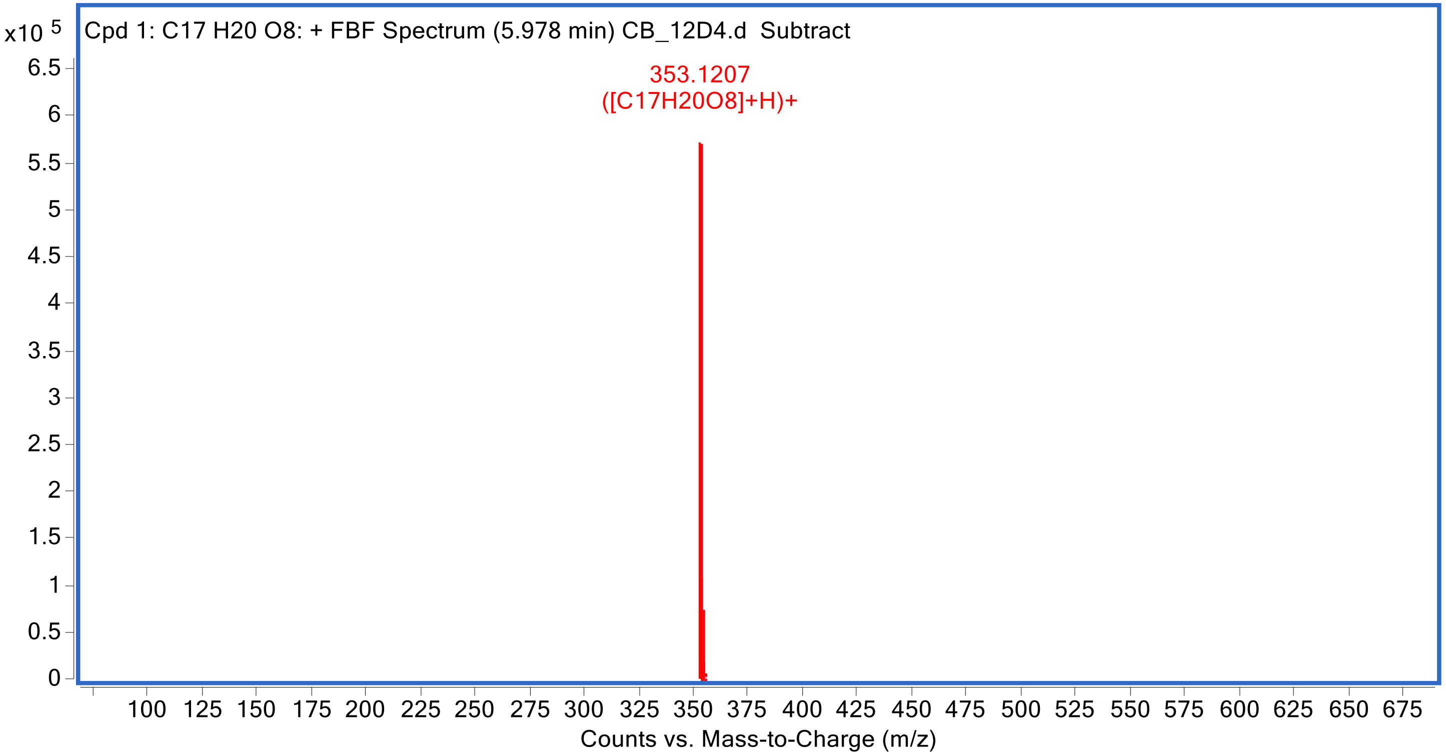

Figure S30. HR-ESI-MS of compound **4**
